# Membrane-Disruption-Enhanced Synergistic Antimicrobial Action of Essential Oils and ε-Polylysine for Targeted Inhibition of Spoilage Yeasts in Blue Honeysuckle (*Lonicera caerulea* L.)

**DOI:** 10.3390/foods15162784

**Published:** 2026-08-08

**Authors:** Yang Yu, Jiayuan Luo, Mingjie Jia, Yihong Bao

**Affiliations:** 1 College of Food and Health, Northeast Forestry University, Harbin 150040, China; yuyang20010214@163.com (Y.Y.); ljy199636@nefu.edu.cn (J.L.); jmj062626@163.com (M.J.); 2 Key Laboratory of Forest Food Resources Utilization of Heilongjiang Province, Harbin 150040, China

**Keywords:** essential oil, ε-polylysine, synergistic antimicrobial action, spoilage yeasts, blue honeysuckle

## Abstract

The spoilage of blue honeysuckle during storage is mainly caused by specific dominant microorganisms, while most antimicrobial systems are developed without considering these target spoilage species. In this study, dominant spoilage yeasts were isolated from blue honeysuckle berries, and an antimicrobial system was constructed using these yeasts, along with common foodborne bacteria as target strains. The results showed that the antimicrobial system exhibited significant synergistic antimicrobial activity. Further analysis indicated that the essential oils disrupted the cell membrane structure and increased membrane permeability, thereby promoting the interaction of ε-PL with intracellular components. Multiple physiological alterations were detected after treatment, including membrane depolarization, leakage of intracellular components, accumulation of intracellular reactive oxygen species, and suppression of cellular metabolism. These observations suggested that damage to microbial cells might be synergistically induced by the composite system. Application of the system to blue honeysuckle berries effectively inhibited the growth of endogenous spoilage microorganisms and delayed quality deterioration during storage, and significantly reduced weight loss and decay rates, as well as changes in color, anthocyanin content, and vitamin C content. These results indicate that constructing antimicrobial systems based on dominant spoilage microorganisms can improve preservation efficiency and provide a useful approach for the storage of blue honeysuckle and similar fruits.

## 1. Introduction

The spoilage of fresh fruits during postharvest storage is primarily driven by the growth of specific dominant microorganisms rather than the entire microbial community. Blue honeysuckle (*Lonicera caerulea* L.), a berry fruit rich in anthocyanins and other bioactive compounds, is known as the “king of the third generation small berries” [1]. Furthermore, blue honeysuckle possesses significant medicinal value, including antioxidant, anti-inflammatory and antimicrobial activities. However, postharvest blue honeysuckle exhibits high moisture content and vigorous metabolism, and it is particularly susceptible to microbial contamination, resulting in softening, rot, and significant loss of nutritional quality. Dominant spoilage yeasts isolated from blue honeysuckle berries were identified as key contributors to fruit deterioration. Therefore, controlling these key spoilage microorganisms is essential for improving the preservation efficiency of blue honeysuckle.

At present, commonly used preservation strategies for fruits mainly rely on chemical preservatives and low-temperature storage. Although chemical preservatives are effective at inhibiting microbial growth, their potential safety concerns and residues have limited their use [2]. Low-temperature storage can retard microbial growth and fruit respiration, but it requires strict temperature control and may induce chilling injury, thereby affecting fruit quality. In recent years, natural antimicrobial agents have attracted increasing attention as safer alternatives for fruit preservation. However, most existing antimicrobial systems are designed for broad-spectrum activity without considering the specific microorganisms responsible for fruit spoilage. This lack of targeted antimicrobial design often leads to suboptimal preservation effects and inefficient use of antimicrobial agents.

Essential oils are widely recognized as promising natural antimicrobial substances due to their broad-spectrum activity and low toxicity. *Litsea cubeba* essential oil (LCEO) is a natural volatile oil extracted from *Litsea cubeba* fruit, which has been demonstrated to possess diverse biological activities, including antioxidant, antimicrobial, and anti-inflammatory properties [3]. It has been reported that when cherry tomatoes were treated with LCEO nanoemulsion for 9 min, the number of *Salmonella* was reduced by 6.50 ± 0.20 log CFU/mL [4]. Cinnamon essential oil (CEO), a volatile oil extracted from cinnamon bark, exhibits functional properties, including antioxidant, anti-inflammatory, antitumor and antimicrobial activities. It has been classified as a recognized safe food additive by the U.S. Food and Drug Administration (FDA) [5]. It has been reported that adding CEO extended the shelf life of mangoes by 40%, whereas lemongrass essential oil extended it by only 25% at the same dosage [6]. However, the practical application of essential oils is restricted by their poor water solubility, high volatility, and instability, which limit their persistence and effectiveness in food systems. On the other hand, ε-polylysine (ε-PL), a natural cationic antimicrobial peptide, has been widely used in the food industry because of its safety and stability. It exerts antimicrobial activity primarily through electrostatic interactions with negatively charged cell surfaces, leading to membrane damage and inhibiting microbial growth. Nevertheless, ε-PL alone may not achieve sufficient antimicrobial efficacy against certain spoilage microorganisms.

Combining different natural antimicrobial agents has been considered an effective strategy to enhance antimicrobial performance through synergistic effects. In particular, essential oils and ε-PL may exhibit complementary mechanisms: essential oils can disrupt cell membrane structure and increase permeability, while ε-PL can further interact with intracellular components after penetrating the cell membrane. Despite this potential, the mechanism of synergy between essential oils and ε-PL has not been clearly elucidated, particularly in the context of targeted inhibition of dominant spoilage microorganisms in specific fruit systems, such as blue honeysuckle.

Therefore, in this study, dominant spoilage yeasts were isolated from blue honeysuckle berries and used as target strains. An antimicrobial system composite essential oil–polylysine (EOS-PL) based on *Litsea cubeba* essential oil, cinnamon essential oil, and ε-polylysine was constructed to achieve targeted inhibition. The antimicrobial activity and synergistic effects of EOS-PL were systematically evaluated, and its mechanism was investigated with emphasis on membrane disruption and intracellular responses. In addition, the system’s application for blue honeysuckle preservation was assessed to verify its practical effectiveness. This study aims to provide a basis for developing targeted antimicrobial strategies for fruit preservation.

## 2. Materials and Methods

### 2.1. Materials and Reagents

The blue honeysuckle variety selected for this experiment is “Lanjingling”, harvested in June 2025 from a berry plantation in Harbin, China (126°56′2″ E, 45°46′10″ N). Fresh berries of uniform size and 80% ripeness that were plump and compact, without mechanical damage or pests and diseases, were selected. After harvesting, they were carefully stored in plastic food boxes and transported back to the Food Microbiology Laboratory of Northeast Forestry University within 2 h. *E. coli* (ATCC25922) and *S. aureus* (ATCC29213) were preserved in the Food Microbiology Laboratory of Northeast Forestry University. *Litsea cubeba* essential oil (LCEO, ≥75%), Cinnamon essential oil (CEO, ≥85%), ε-polylysine (ε-PL, ≥95%), DiBAC_4_(3), DCFH-DA and YPD medium were purchased from Shanghai Yuanye Bio-Technology Co., Ltd. (Shanghai, China). The chemical compositions of LCEO and CEO have been included in the Supplementary Materials. The SYTO9/PI staining kit was purchased from Bjbalb Technology Co., Ltd. (Beijing, China). Glutaraldehyde was obtained from Aladdin Biochemical Technology Co., Ltd. (Shanghai, China). The alkaline phosphatase (AKP) assay kit, ATP content assay kit, and BCA kit were purchased from Nanjing Jiancheng Bioengineering Institute (Nanjing, China).

### 2.2. Isolation and Identification of Spoilage Yeasts

Fresh blue honeysuckle berries at the early natural decay stage were selected, rinsed with distilled water and air-dried. Decayed tissues were aseptically collected and immersed in sterile normal saline, then incubated and shaken at 180 r/min and 28 °C for 30 min. The mixture was centrifuged at 5000× *g* for 5 min, and the supernatant was harvested as the original microbial suspension. One milliliter of the original suspension was inoculated into sterile YPD liquid medium and cultured at 28 °C with shaking for 18 h. The cultured broth was serially diluted, and 0.1 mL of the dilution at the appropriate concentration was spread evenly onto YPD solid plates. All plates were incubated inverted at 28 °C for 18–24 h. Dense colonies were picked and subjected to streak purification until pure single colonies were obtained. Two dominant spoilage yeasts were isolated according to colony occurrence frequency and designated as Yeast C and Yeast R, respectively. Morphological characteristics of the isolates were observed under a light microscope (BA310 Digital, Motic China Group Co., Ltd., Xiamen, China). The two isolates were then sent to Sangon Biotech (Shanghai) Co., Ltd. (Shanghai, China) for molecular identification, and a phylogenetic tree was constructed using MEGA 11.0 (Mega Software, Tempe, AZ, USA).

### 2.3. Synergistic Antimicrobial Effect of Composite Essential Oil (EOS) and ε-PL

#### 2.3.1. Determination of MIC of Single Antimicrobial Agent (SA)

Based on previous research and with slight modifications, the minimum inhibitory concentrations (MICs) were determined using the microbroth dilution method [7]. LCEO and CEO were dissolved in 1% Tween 80, while ε-PL was dissolved in sterile water and added to microbial suspensions with a concentration of 1 × 10^6^ CFU/mL, resulting in final concentrations of 4.000, 2.000, 1.000, 0.500, 0.250, 0.125, 0.063, and 0.031 mg/mL, respectively. Yeasts were cultured at 28 °C for 18 h, and bacteria were cultured at 37 °C for 24 h. The lowest concentration at which no visible growth was observed was recorded as the MIC value.

#### 2.3.2. Determination of MIC of EOS

LCEO and CEO were added to a microbial suspension with a concentration of 1 × 10^6^ CFU/mL in ratios of 1:1, 1:2, and 2:1 (*v*/*v*), respectively. The remaining procedures were the same as Section 2.3.1, and the MIC of EOS was measured.

#### 2.3.3. Determination of MIC of EOS-PL

EOS and ε-PL were added to a microbial suspension with a concentration of 1 × 10^6^ CFU/mL in ratios of 1:1, 1:2, and 2:1 (*w*/*w*), respectively. The remaining procedures were the same as Section 2.3.1, and the MIC of EOS-PL was measured.

#### 2.3.4. Determination of Fractional Inhibitory Concentration Index (FICI)

The Fractional Inhibitory Concentration Index (FICI) is used to evaluate the synergistic antimicrobial effect of EOS and ε-PL. It was determined using the checkerboard dilution method, as reported by Li [8]. A sterile 96-well plate was employed. Columns 1 to 6 were filled with 50 μL of EOS serially diluted from 2 to 1/16 MIC. Similarly, rows A to F were filled with 50 μL of ε-PL serially diluted from 2 to 1/16 MIC. Subsequently, 100 μL of the microbial suspension was added to each well. Yeasts were cultured at 28 °C for 18 h, and bacteria were cultured at 37 °C for 24 h. The result is based on the following formula:

FICI = MIC_(EOS/EOS+ε-PL)_/MIC_EOS_ + MIC_ε-PL/EOS+ε-PL)_/MIC_ε-PL_

where MIC_(EOS/EOS+ε-PL)_ and MIC_(ε-PL/EOS+ε-PL)_ were the effective concentrations of compound EOS and ε-PL in the combinations, respectively (mg/mL).

FICI ≤ 0.5 was indicated as synergism; 0.5 < FICI ≤ 1 was indicated as an additive effect; 1 < FICI ≤ 2 was indicated as indifference; FICI > 2 was indicated as antagonism.

### 2.4. Determination of Antimicrobial Activity of EOS-PL

According to the results of Section 2.3, EOS and ε-PL have a good synergistic antimicrobial effect, and the antimicrobial effect is best when EOS (LCEO:CEO = 1:2): ε-PL = 2:1. Therefore, this ratio was chosen for further research. Based on previous studies, the growth curves of tested strains were determined to evaluate the antimicrobial activity of EOS-PL [9]. The OD_600_ of the microbial suspension was adjusted to 0.1. EOS-PL was added to the microbial suspension at final concentrations of 2 MIC, MIC, and 1/2 MIC, with the group without EOS-PL as the control. The samples were cultured at 37 °C and 28 °C for 24 h, and their growth was measured every 2 h at 600 nm using a multimode microplate reader (Multiskan ET, Thermo Fisher Scientific, Waltham, MA, USA), and growth curves were plotted.

### 2.5. Determination of Antimicrobial Mechanism of EOS-PL

#### 2.5.1. Scanning Electron Microscopy (SEM) Examination

SEM was performed following the method of Sun [10]. EOS-PL was added to the microbial suspension to achieve mass concentrations of 2 MIC, MIC, and 1/2 MIC, respectively, with the group supplemented with an equal volume of sterile PBS serving as the control. The mixtures were incubated in a shaker at 200 rpm for 4 h, and microbial precipitates were collected by centrifugation. The precipitates were washed three times with sterile PBS, followed by the addition of 2.5% glutaraldehyde, and fixed at 4 °C overnight. Microbial cells were collected by centrifugation, washed three times with PBS, and the precipitate was collected by centrifugation. A graded ethanol series was used for dehydration: the cells were treated sequentially with 30%, 50%, 70%, 90%, and 100% (*v*/*v*) ethanol solutions. Subsequently, the samples were washed twice with isoamyl acetate. Finally, the dehydrated samples were freeze-dried under vacuum. The prepared specimens were observed using a scanning electron microscope (Apreo S HiVac, Thermo Fisher Scientific, USA).

#### 2.5.2. Measurement of Cell Membrane Potential

The 4 test strains were cultured to the logarithmic growth phase, centrifuged at 4000× *g* for 5 min, washed three times with 0.1 mol/L PBS buffer (pH = 7.4), and then resuspended in PBS solution to adjust the OD_600_ to 0.1. Subsequently, EOS-PL was added to the microbial suspension to achieve mass concentrations of 2 MIC, MIC, and 1/2 MIC, respectively, with the group supplemented with an equal volume of sterile PBS serving as the control. The mixtures were incubated in a shaker at 200 rpm for 4 h, and the microbial cells were collected by centrifugation and washed twice with PBS. DiBAC_4_(3) was added to the suspensions to a final concentration of 2.5 μg/mL. The stained suspensions were incubated in darkness for 30 min. After that, the samples were centrifuged again and resuspended in PBS. Fluorescence was observed using a fluorescence microscope (Axio Imager A2, ZEISS, Oberkochen, Germany) at an excitation wavelength of 488 nm and an emission wavelength of 525 nm [11].

#### 2.5.3. Measurement of Intracellular Reactive Oxygen Species (ROS)

The ROS levels were detected following the method of Cui [12]. EOS-PL was added to the microbial suspension to achieve mass concentrations of 2 MIC, MIC, and 1/2 MIC, respectively, with the group supplemented with an equal volume of sterile PBS serving as the control. After incubation in a shaker at 200 rpm for 4 h, the mixtures were centrifuged at 5000× *g* for 5 min, and the supernatant was discarded. The pellets were washed with sterile PBS, then incubated with 10 μM DCFH-DA in the dark for 30 min. After that, the pellets were washed three times with sterile PBS, and fluorescence was observed using a fluorescence microscope at an excitation wavelength of 488 nm and an emission wavelength of 525 nm.

#### 2.5.4. Measurement of Cell Membrane Integrity

The 4 test strains were cultured to the logarithmic growth phase, centrifuged at 4000× *g* for 5 min, washed three times with 0.1 mol/L PBS buffer (pH = 7.4), and then resuspended in PBS solution to adjust the OD_600_ to 0.1. Subsequently, EOS-PL was added to the microbial suspension to achieve mass concentrations of 2 MIC, MIC, and 1/2 MIC, respectively, with the group supplemented with an equal volume of sterile PBS serving as the control. The mixtures were incubated in a shaker at 200 rpm for 4 h, and the microbial cells were collected by centrifugation and washed twice with PBS. PI (12 μM) and SYTO-9 (2 μM) were added to the suspension, and the mixture was gently pipetted to achieve homogeneity. It was then incubated at room temperature in the dark for 30 min. After incubation, the mixture was centrifuged, and the supernatant was discarded. The resulting pellet was washed and resuspended in sterile PBS, and fluorescence was observed using a fluorescence microscope at an excitation wavelength of 488 nm and an emission wavelength of 635 nm [13].

#### 2.5.5. Measurement of Cell Membrane Permeability

Cell membrane permeability was evaluated by measuring the extracellular relative conductivity (RC) according to the method described by Wang et al. [14]. The 4 test strains were cultured to the logarithmic growth phase, centrifuged at 5000× *g* for 5 min, then washed and resuspended in a 5% sterile glucose solution. This process was repeated until the sample solution’s conductivity approached that of the 5% glucose solution, thereby making it an isotonic microbial suspension. EOS-PL (2 MIC, 1 MIC, 1/2 MIC, and 0) was added to the 5% glucose solution, and its conductivity was measured using a conductivity meter (DDS-11A, Shanghai Yidian Scientific Instrument Co., Ltd., Shanghai, China) and recorded as L1. EOS-PL was then added to the isotonic microbial suspension, followed by a 12 h incubation. The conductivity of the mixture was measured every 2 h and recorded as L2. For the control group, the conductivity of the microbial suspension in 5% glucose heated in a boiling water bath for 5 min was determined and labeled as L0. RC is calculated as follows:

RC (%) = 100 × (L_2_ − L_1_)/L_0_

#### 2.5.6. Measurement of Leakage of Cellular Contents

Leakage of cellular contents was evaluated using a previously described method with slight modifications [15]. EOS-PL was added to the microbial suspension to achieve mass concentrations of 2 MIC, MIC, and 1/2 MIC, respectively, with the group supplemented with an equal volume of sterile PBS serving as the control. After incubation in a shaker at 200 rpm for 4 h, the mixtures were centrifuged at 5000× *g* for 10 min, and the supernatants were collected for analysis. Protein and nucleic acid leakage was measured at 280 nm and 260 nm using a microplate reader. Alkaline phosphatase (AKP) activity was determined using an AKP assay kit, and ATP concentration was measured using an ATP content assay kit.

#### 2.5.7. SDS-PAGE

The analysis of SDS-PAGE was performed according to a previously reported method with slight modifications [16]. EOS-PL was added to the microbial suspension to achieve mass concentrations of 2 MIC, MIC, and 1/2 MIC, respectively, with the group supplemented with an equal volume of sterile PBS serving as the control. After incubation in a shaker at 200 rpm for 4 h, the mixtures were centrifuged at 5000× *g* for 5 min, the supernatant was discarded, and the pellets were washed with sterile PBS. The cells were disrupted by ultrasonication (disruption conditions: working for 5 s, intervals of 15 s, total duration of 10 min, power of 400 W). After ultrasonication, the mixtures were centrifuged at 5000× *g* for 10 min, and the supernatant was collected. The protein concentration in the supernatant was determined using a BCA kit, and the protein concentration of each group was adjusted to ensure consistency. A 5× protein loading buffer was added to the samples, which were then boiled for 5 min. SDS-PAGE was performed using a 12% separating gel and a 5% stacking gel, followed by Coomassie Brilliant Blue staining [17].

### 2.6. Application of EOS-PL in the Preservation of Blue Honeysuckle Berries

Fresh blue honeysuckle berries with uniform color and size were selected. After cleaning the surface dirt with distilled water, the berries were soaked in 0.2% NaClO solution to eliminate surface microorganisms, then rinsed again with sterile distilled water and air-dried at room temperature. The samples were divided into 4 groups in total. The first group, with no treatment applied, was designated as the control group; the second group of samples was soaked in EOS solution with a concentration of MIC for 30 s and labeled as the EOS group; the third group of samples was soaked in ε-PL solution with a concentration of MIC for 30 s and designated as the PL group; the fourth group of samples was soaked in EOS-PL solution with a concentration of MIC for 30 s and recorded as the EOS-PL group.

#### 2.6.1. Appearance and Color

The appearance changes of blue honeysuckle berries were captured using a mobile phone, and color determination was performed using a colorimeter (CR-10 plus, Konica Minolta, Tokyo, Japan). For each group of samples, the lightness value (L*), redness value (a*), and yellowness value (b*) were measured separately, and the total color difference (△E) was calculated as follows:

$$\Delta E\;=\;\sqrt{{(L^{*}\;-\;L)}^{2}+\;{(a^{*}-\;a)}^{2}\;+\;{(b^{*}\;-\;b)}^{2}}$$

where L*, a*, and b* were the color values of blue honeysuckle berries after being stored for a specific period, while L, a, and b were the color values of initially fresh blue honeysuckle berries.

#### 2.6.2. Weight Loss Rate and Decay Rate

During the storage period, the weight and number of decayed berries in each group were recorded every 2 d, and the weight loss rate and decay rate were calculated as follows:

$$\mathrm{Weight}\;\mathrm{loss}\;\mathrm{rate}\;(\%)\;=\;\frac{m_{0}-m_{1}}{m_{0}}\times 100$$

$$\mathrm{Decay}\;\mathrm{rate}\;(\%)\;=\;\frac{X_{1}}{\;X_{2}}\times 100$$

where m_0_ and m_1_, respectively, represented the initial mass and the mass after storage of blue honeysuckle berries (g); X_1_ and X_2_, respectively, represented the number of decayed and the total number of blue honeysuckle berries.

#### 2.6.3. Determination of pH and Total Soluble Solids (TSS)

An equal mass of blue honeysuckle berries from each treatment group was weighed and ground into a homogenate using a mortar. The homogenate was filtered through 4 layers of cheesecloth to obtain the filtrate. The pH of the filtrate was determined using a pH meter (PHSJ-6L, Shanghai Yidian Scientific Instrument Co., Ltd., Shanghai, China). The TSS content was measured using a digital refractometer (PAL-1, Atago, Tokyo, Japan).

#### 2.6.4. Determination of Anthocyanin, Total Phenols, and Vitamin C (VC) Content

The anthocyanin content was determined using the pH differential method according to the procedure described by Ma et al. [18]. The results were expressed as milligrams of cyanidin-3-glucoside equivalents per 100 g of fresh weight (mg·100 g^−1^ FW).

The total phenol content was determined using the Folin–Ciocalteu method according to Yang et al. [19]. The results were expressed as milligrams of gallic acid equivalent per gram of fresh weight (mg·g^−1^ FW).

The VC content was measured by 2,6-dichlorophenolindophenol titration according to the method described by Yi et al. [20]. The results were calculated as milligrams of ascorbic acid per 100 g of fresh weight (mg·100 g^−1^ FW).

#### 2.6.5. Determination of Malondialdehyde (MDA) Content

The MDA content was determined by the thiobarbituric acid method, and the results were expressed as μmol·g^−1^ FW [21].

#### 2.6.6. Determination of Microbial Index

Based on the method of de Oliveira et al. [22], 25 g of berries were weighed from each group and mixed with 225 mL of sterile physiological saline, then homogenized under sterile conditions. After being cultured at 37 °C for 48 h, the bacterial count was determined using Petrifilm™ colony count test strips (6406, Neogen, Lansing, MI, USA). After being cultured at 28 °C for 5 d, mold and yeast count was determined using Petrifilm™ mold/yeast test strips (6417, Neogen, USA).

### 2.7. Statistical Analysis

All experiments were performed at least three times. Data were presented as mean ± standard deviation (SD) and analyzed using SPSS 25.0 (SPSS Inc., Chicago, IL, USA). Statistical comparisons between groups were performed using one-way analysis of variance (ANOVA), and Duncan’s multiple range test for statistical comparisons was used. A significance level of *p* < 0.05 was used to determine statistical significance.

## 3. Results and Discussion

### 3.1. Morphological and Molecular Biology Identification of Spoilage Yeasts

The dominant spoilage yeasts, *C. krusei* (BYkrnzF01) and *R. mucilaginosa* (BYjhF01), were isolated from decayed blue honeysuckle berries. The morphological and molecular biology identification results are provided in the Supplementary Materials.

### 3.2. Synergistic Antimicrobial Effect of EOS and ε-PL

MIC is a key indicator for evaluating antimicrobial activity. The MIC results of SA, EOS, and EOS-PL are shown in Table 1. The MIC of LCEO against *R. mucilaginosa* and *E. coli* was 0.500 mg/mL, indicating strong antimicrobial activity. A significant inhibitory effect of LCEO against Gram-negative bacteria was reported by Yang et al. [23], with the MIC against *E. coli* determined to be less than 1.000 mg/mL, which was consistent with the findings of the present study. This indicated that LCEO can act on the phospholipid bilayer of the cell membrane, and membrane integrity was consequently disrupted, thereby leading to strong inhibitory effects on Gram-negative bacteria. CEO exhibited relatively good inhibitory effects against the yeasts *C. krusei* and *R. mucilaginosa*. This was attributed to the targeted binding between cinnamaldehyde, the main active component of CEO, and ergosterol, resulting in the impairment of cell membrane function [24]. The MIC of ε-PL against Gram-negative bacteria, Gram-positive bacteria and yeasts was determined to be 1.0000–2.000 mg/mL, slightly higher than that of LCEO and CEO. According to a previous study, the MIC of ε-PL against *S. aureus* was 1.000 mg/mL, which differs slightly from this study [25]. This variation was attributed to differences in the initial inoculum concentrations of the strains.

The optimal antimicrobial activity of EOS was observed at a ratio of LCEO to CEO of 1:2. The MIC against *C. krusei*, *R. mucilaginosa*, *E. coli*, and *S. aureus* was 0.250, 0.125, 0.125, and 0.500 mg/mL, respectively, all of which were lower than that of SA. Liu et al. [26] reported that the combined application of LCEO and CEO significantly enhanced the antimicrobial activity against A. flavus (*p* < 0.05), which was similar to the results of this study. As shown in Table 1, the most potent antimicrobial activity of EOS-PL was observed at a ratio of EOS (LCEO:CEO = 1:2) to ε-PL of 2:1. The MICs against *C. krusei*, *R. mucilaginosa*, *E. coli* and *S. aureus* were 0.125, 0.063, 0.063 and 0.125 mg/mL, respectively, which were lower than those of EOS and ε-PL used alone.

Synergistic antimicrobial activity is defined as the combined inhibitory effect of two or more antimicrobial agents exceeding the sum of their individual effects when used separately. In this study, the synergistic antimicrobial effect of EOS and ε PL was evaluated using FICI values. As shown in Table 1, when EOS:ε-PL = 2:1, the FICI values of EOS PL against *C. krusei*, R. mucilaginosa, *E. coli*, and *S. aureus* were no higher than 0.500, indicating that the combination of EOS and ε-PL exerted a significant synergistic antimicrobial effect against these 4 strains. This was attributed to the main active components of EOS, including cinnamaldehyde and citral, disrupting the microbial cell membrane structure and increasing its permeability, thereby facilitating the entry of ε-PL into damaged cells through electrostatic interactions and ultimately enhancing antimicrobial efficacy. Gao reported that ε-PL combined with carvacrol exhibited a synergistic effect against *E. coli* and *S. aureus*, with FICI values of 0.375 and 0.500, respectively, which was consistent with the findings of this study [27].

### 3.3. Antimicrobial Activity of EOS-PL

**Figure 1:**
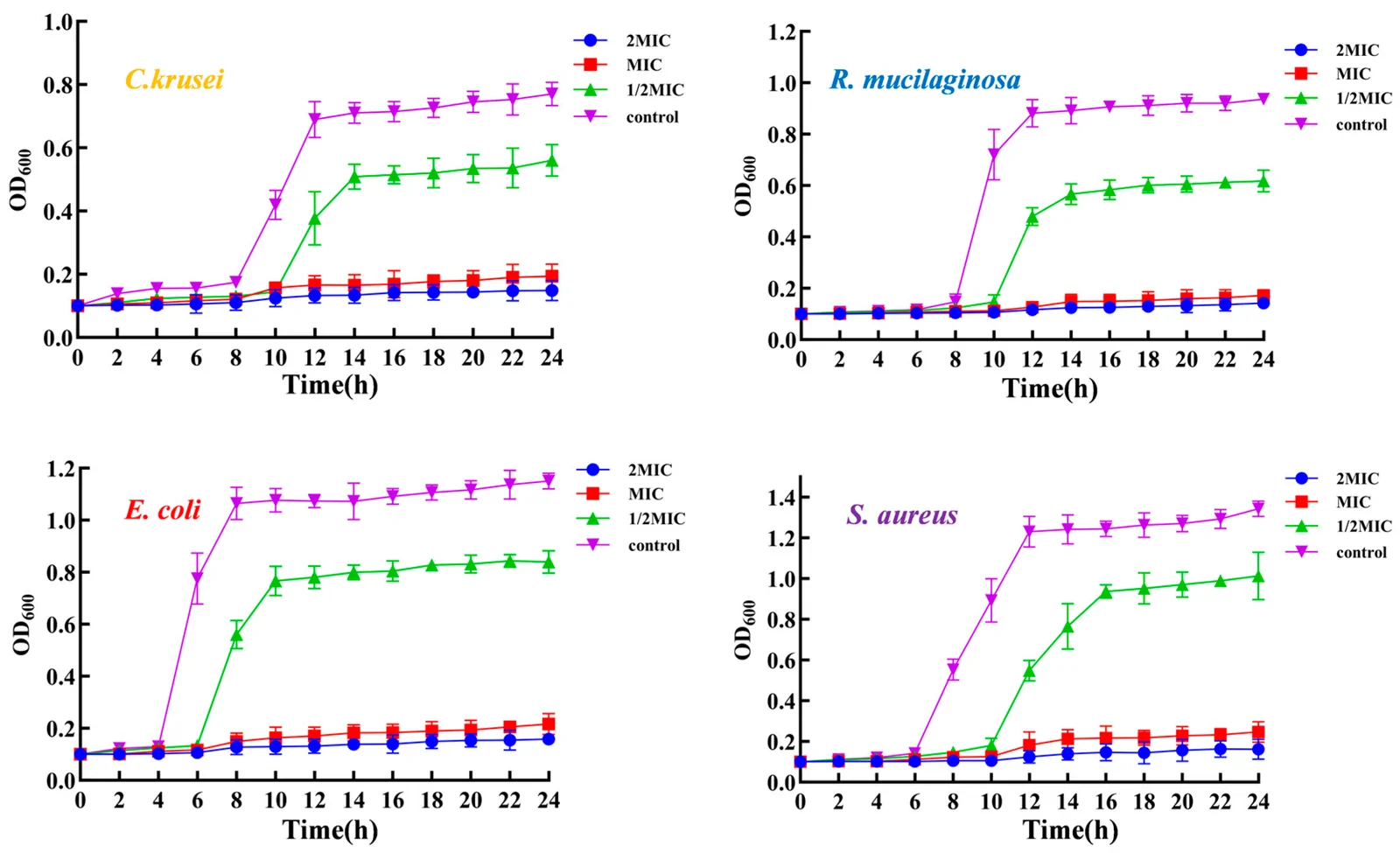
Effects of different concentrations of EOS-PL on the growth curves of tested strains. *n* = 3.

The growth curves of *C. krusei*, *R. mucilaginosa*, *E. coli*, and *S. aureus* under different concentrations of EOS-PL are shown in Figure 1. The control groups were characterized by a typical “s” shape, exhibiting distinct logarithmic and stationary phases. The logarithmic phase was observed at 8–12 h for *C. krusei* and *R. mucilaginosa*, 4–8 h for *E. coli*, and 6–12 h for *S. aureus*, after which all four strains entered the stationary phase.

When treated with 1/2 MIC EOS-PL, the growth of all test strains was inhibited. The logarithmic phase was delayed by approximately 2 h for *C. krusei*, *R. mucilaginosa*, and *E. coli*, and by 4 h for *S. aureus*. This variation could be attributed to differences in adaptive responses among microbial species to the gradual release of essential oil components. At EOS-PL concentrations of MIC and 2 MIC, no significant changes in OD_600_ were observed over time. However, the maximum biomass was significantly reduced (*p* < 0.05) compared to the control group, indicating nearly complete inhibition of microbial growth. These results demonstrated that EOS-PL exhibited inhibitory effects against Gram-positive bacteria, Gram-negative bacteria, and yeasts, with efficacy positively correlated with concentration.

### 3.4. Antimicrobial Mechanism of EOS-PL

#### 3.4.1. Effect of EOS-PL on Microbial Morphology

**Figure 2:**
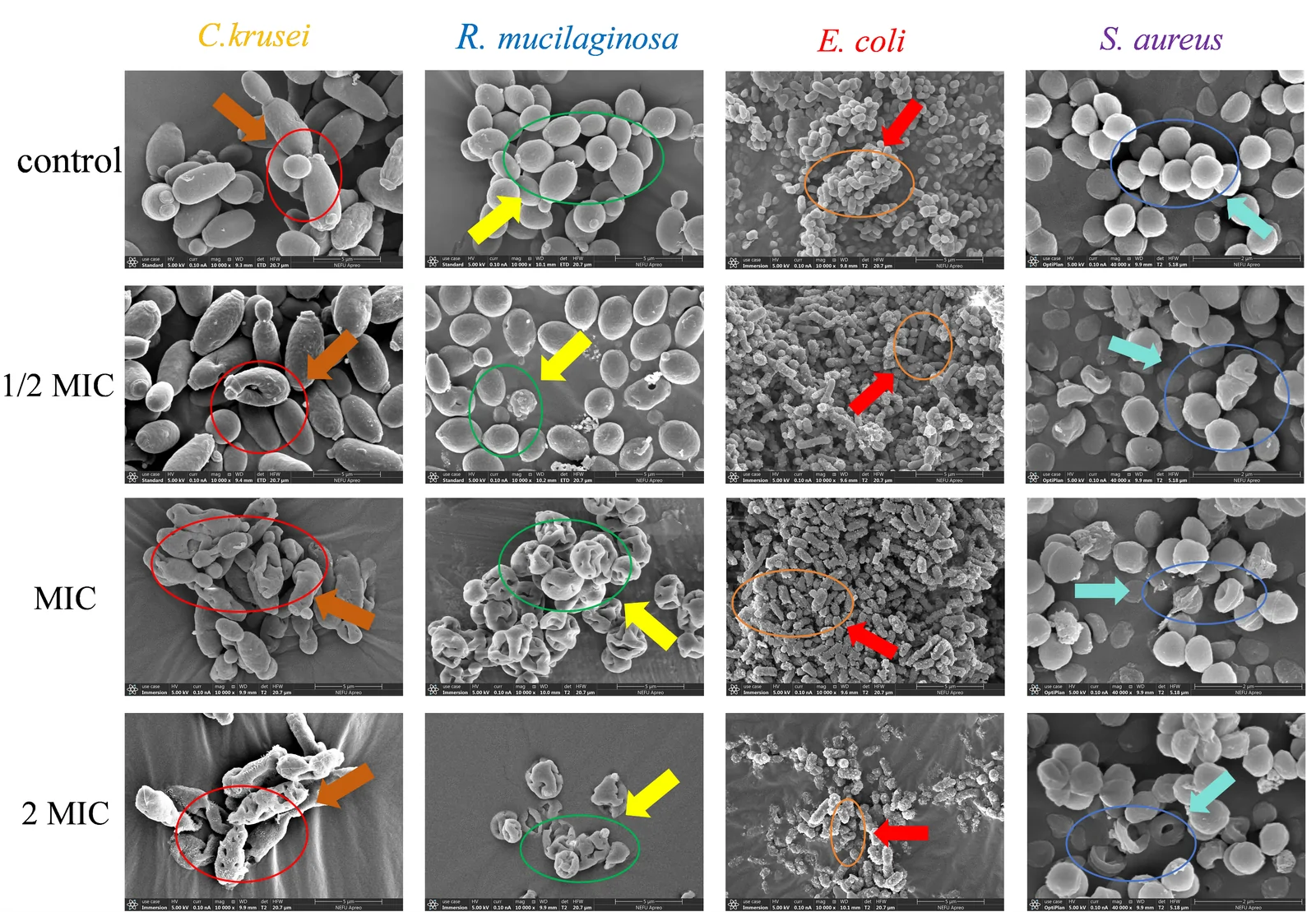
Effects of different concentrations of EOS-PL on the surface morphology of the tested strains. The circles/arrows of different colors highlight the morphological changes of different strains.

The effects of EOS-PL treatments at different concentrations on the morphologies of the four tested strains are shown in Figure 2. Untreated microbial cells were intact, had smooth surfaces, and were more rounded and plump than those treated with EOS-PL. For *C. krusei*, the microbial cells in the control group had smooth surfaces and exhibited an intact elliptical shape. In the 1/2 MIC group, concavities began appearing on the microbial cells. In the MIC group, the surface roughness of the microbial cells increased, and the degree of cell membrane damage was aggravated. In the 2 MIC group, the microbial cells were severely damaged, with numerous holes appearing in the field of view and a loss of their normal shape. For *R. mucilaginosa*, the microbial cells in the control group were plump and rounded, maintaining a good cellular structure. In the 1/2 MIC group, shrinkage began on cell surfaces, and the cells became irregularly shaped. In the MIC group, obvious concavities appeared on the microbial cells, accompanied by protoplast leakage. In the 2 MIC group, the microbial cells were severely damaged and difficult to maintain normal morphology.

For *E. coli*, the microbial cells in the control group had smooth surfaces and maintained intact rod-shaped structures. In the 1/2 MIC group, slight shrinkage and limited leakage of intracellular materials were noted in some cells. In the MIC group, the bacterial cells were severely damaged and suffered severe deformation, with holes appearing in some cells. In the 2 MIC group, the microbial cells basically lost their rod-shaped structures. For *S. aureus*, the cells in the control group showed smooth surfaces and typical spherical morphology. In the 1/2 MIC group, minor surface roughness and fine concavities were apparent. In the MIC group, many cells lost their regular spherical shape, and cell rupture along with cytoplasmic leakage was visible. In the 2 MIC group, further destruction of the spherical structure was accompanied by extensive concavities, aggravated leakage, and the formation of numerous pores. In summary, EOS-PL caused severe morphological damage to microbial cells. Liu demonstrated that allicin nanoemulsion and ε-PL exerted a synergistic antimicrobial effect, and the combined treatment also altered the cellular morphology and structure of *E. coli*, which was in accordance with the findings of this study [28].

#### 3.4.2. Effect of EOS-PL on Cell Membrane Potential

DiBAC_4_(3) is an anionic fluorescent dye that can bind to intracellular proteins. When microorganisms are subjected to external stimuli, ions enter or exit the microbial cells, resulting in changes in fluorescence intensity that reflect changes in cell membrane potential.

**Figure 3:**
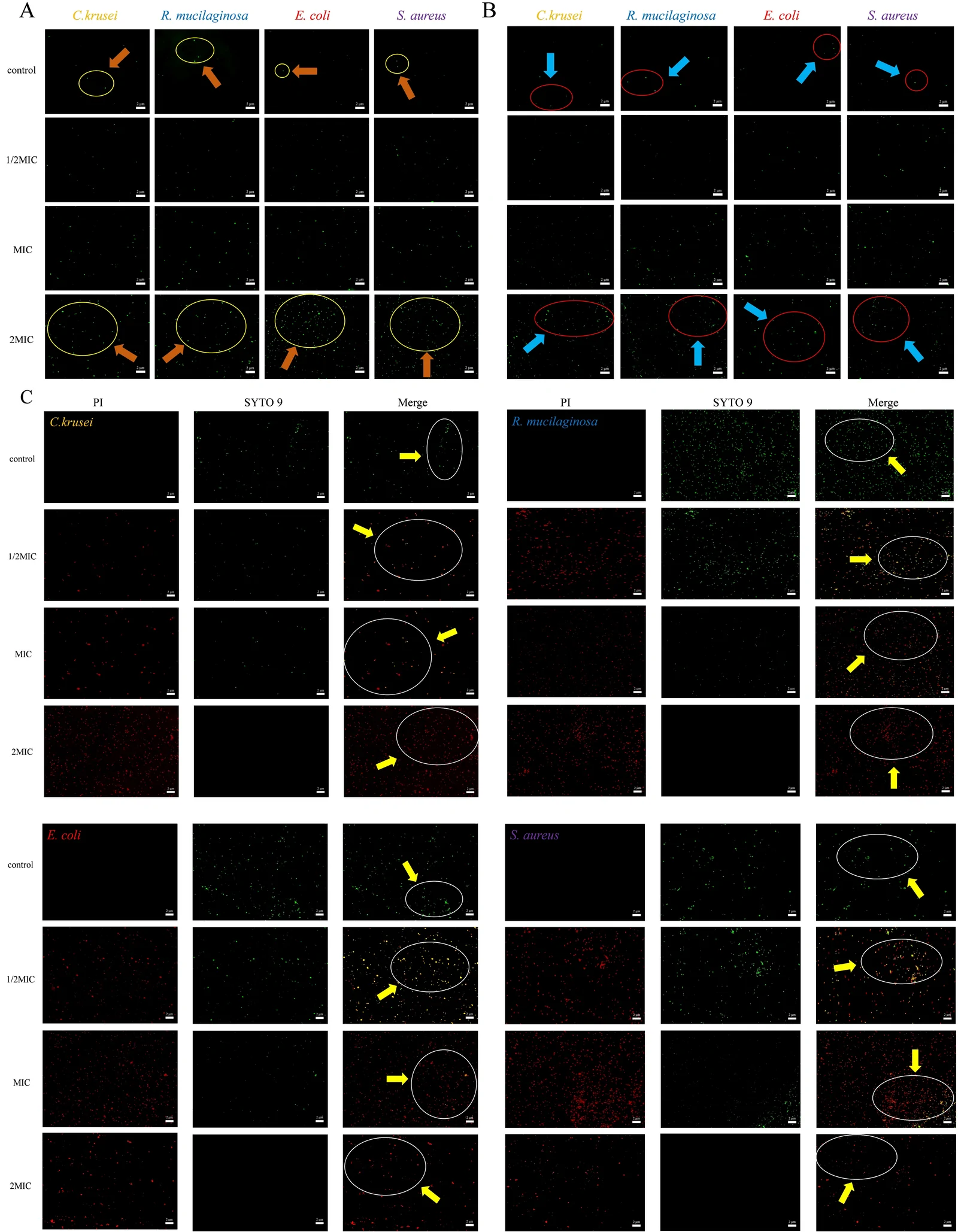
Effects of different concentrations of EOS-PL on the membrane potential (**A**), intracellular ROS levels (**B**), and membrane integrity (**C**) of the tested microbial cells. Different colored circles/arrows highlight the degree of impact of different concentrations of EOS-PL on membrane potential, intracellular ROS levels, and membrane integrity.

The results of this study are shown in Figure 3A. The fluorescence intensity of the four tested strains treated with 2 MIC, MIC, and 1/2 MIC of EOS-PL was higher than that of the control group, and the fluorescence intensity was enhanced with the increase in EOS-PL concentration. It was indicated that EOS-PL might induce depolarization of the tested microbial cells. Depolarized cell membranes will be unable to function effectively as protective barriers; thus, EOS-PL can easily penetrate the depolarized cell membranes to form pores, thereby causing the leakage of intracellular ions [29].

#### 3.4.3. Effect of EOS-PL on Intracellular ROS Levels

ROS are highly reactive chemical substances, which include superoxide anion radicals, hydrogen peroxide, highly reactive hydroxyl radicals, and others. An increase in ROS levels may damage microbial DNA and activate the protease RecA, ultimately leading to programmed cell death [30].

In this study, DCFH-DA fluorescent dye was used to detect ROS in the four tested strains. As shown in Figure 3B, compared with the control group, the intracellular ROS content gradually increased with increasing concentrations of EOS-PL. This result was consistent with that reported by Boda et al. [31]; both of these indicate that increased concentrations of antimicrobial agents might trigger higher levels of oxidative stress in cells, thereby ultimately inhibiting microbial growth.

#### 3.4.4. Effect of EOS-PL on Cell Membrane Integrity

SYTO-9 can penetrate cell membranes, bind to nucleic acids, and emit green fluorescence, while PI can only stain cells with damaged cell membranes and emit red fluorescence. Therefore, the integrity of the cell membranes of the four tested strains after EOS-PL treatment was assessed by double-staining with PI and SYTO-9 fluorescent probes.

The results are shown in Figure 3C; untreated cells exhibited exclusively green fluorescence, indicating intact membrane functionality and structural integrity. When the concentration reached 1/2 MIC, both red and green fluorescence were observed, suggesting that it caused damage to the cell membrane of some microorganisms but did not destroy all microbial cells. At the MIC concentration, red fluorescence became dominant, indicating severe membrane damage in most cells. At 2 MIC, only red fluorescence was detected, confirming complete loss of membrane integrity. These results demonstrated that the degree of cell membrane damage was positively correlated with the concentration of the antimicrobial agent, consistent with the findings of Dai [32]. This study suggested that EOS-PL might compromise cell membrane integrity, interfere with intracellular substance trafficking, and potentially trigger microbial apoptosis.

#### 3.4.5. Effect of EOS-PL on Cell Membrane Permeability

The increase in RC indicates intracellular ion changes, reflecting cell membrane permeability. When antimicrobial agents act on microorganisms, cell membrane permeability increases, leading to non-selective leakage of intracellular electrolytes (such as K^+^ and Mg^2+^). This leads to an increase in the concentration of charged particles in the extracellular solution, which ultimately manifests as a significant rise in conductivity.

**Figure 4:**
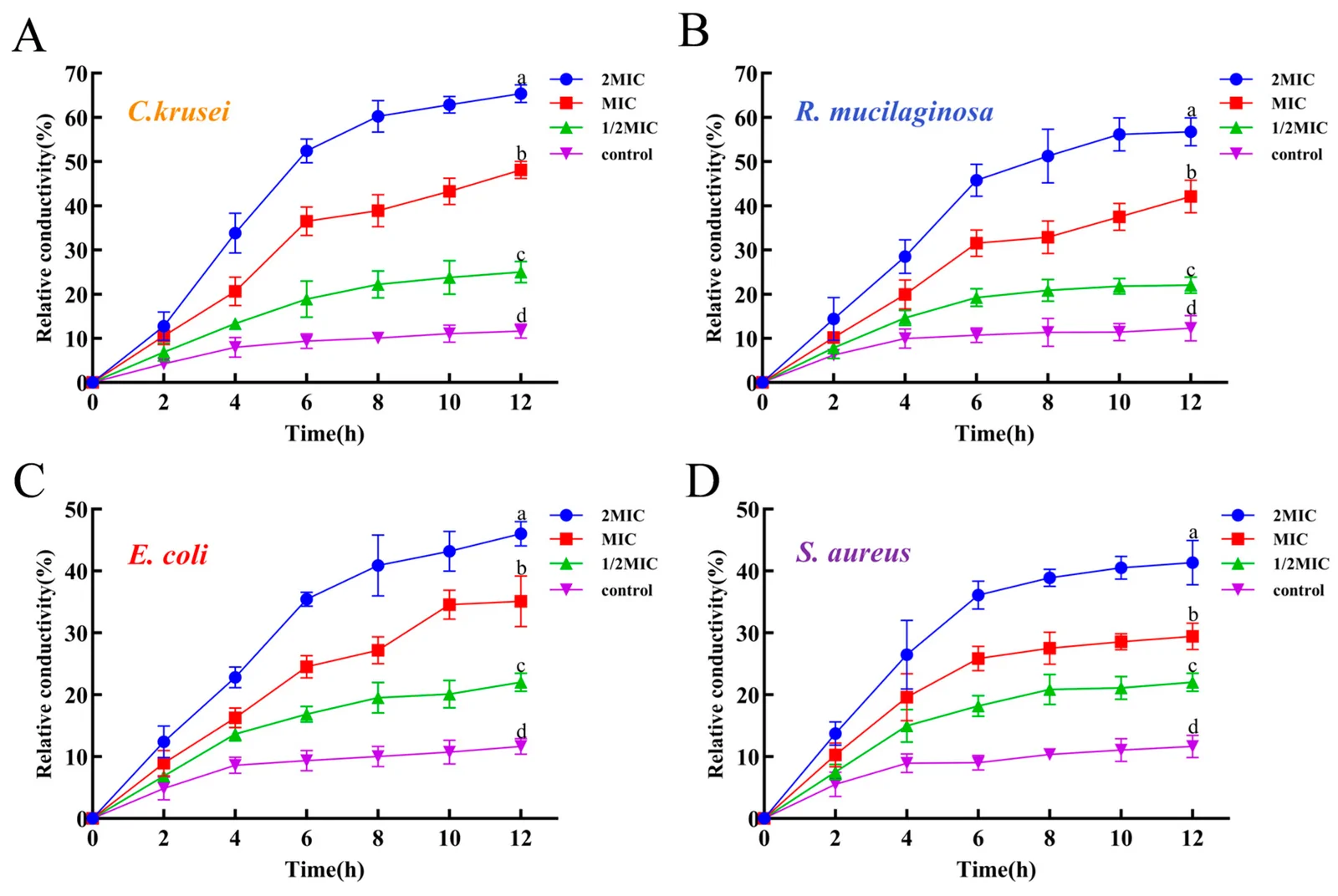
Effects of different concentrations of EOS-PL on the extracellular relative conductivity of *C. krusei* (**A**), *R. mucilaginosa* (**B**), *E. coli* (**C**) and *S. aureus* (**D**). *n* = 3; lowercase letters (a–d) refer to statistically significant differences among the groups (*p* < 0.05).

The results are shown in Figure 4. With the increase in time, the RC of all samples gradually increased. The difference was that the RC of the control group showed a slow upward trend overall, whereas the RC of the EOS-PL treatment groups exhibited an initially fast upward trend that then slowed. Moreover, a significant positive correlation was observed between RC and EOS-PL concentration (*p* < 0.05). The RC results of *C. krusei* are shown in Figure 4A. At 12 h, the RCs of the groups treated with 2 MIC, MIC, and 1/2 MIC were 65.37%, 48.10%, and 25.04%, respectively, which were significantly increased by 461.89%, 313.47%, and 114.90%, respectively, compared with the control group (*p* < 0.05). The results of *R. mucilaginosa* are presented in Figure 4B. At 12 h, the RCs of the groups treated with 2 MIC, MIC, and 1/2 MIC were 56.73%, 42.10%, and 22.07%, respectively, representing significant increases of 360.96%, 242.28%, and 78.85% compared with the control group (*p* < 0.05).

The results of *E. coli* are shown in Figure 4C. At 12 h, the RCs of the groups treated with 2 MIC, MIC, and 1/2 MIC were 46.03%, 35.10%, and 22.00%, respectively, which significantly increased by 295.70%, 201.72%, and 89.11% compared with the control group (*p* < 0.05). The results of *S. aureus* are displayed in Figure 4D. At 12 h, the RCs of the groups treated with 2 MIC, MIC, and 1/2 MIC were 41.37%, 29.43%, and 22.17%, respectively, showing significant increases of 255.59%, 153.01%, and 89.11% compared with the control group (*p* < 0.05). This result was consistent with the findings of Qi [33]; both studies suggest that elevated concentrations of antimicrobial agents tend to increase cell membrane permeability and trigger gradual leakage of intracellular ions, which might consequently raise microbial mortality.

#### 3.4.6. Effect of EOS-PL on Leakage of Intracellular Contents

Leakage of intracellular contents is an important indicator of the extent of cell membrane damage. Proteins and nucleic acids jointly dominate the transmission of genetic information, the construction of cellular structures, and metabolic processes in microorganisms. Once both are damaged, microorganisms will lose their viability.

**Figure 5:**
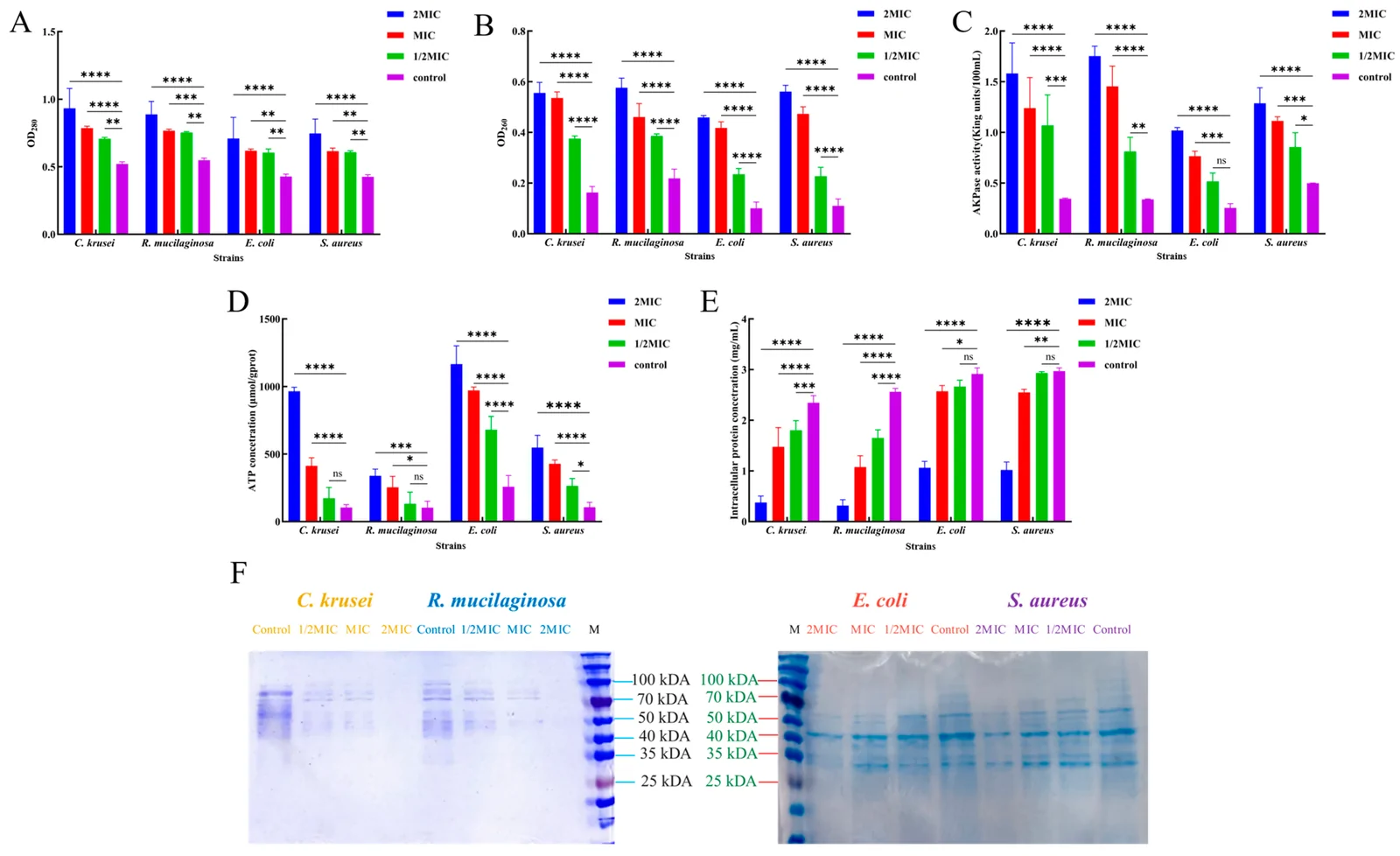
Effects of different concentrations of EOS-PL on protein leakage (**A**), nucleic acid leakage (**B**), AKPase activity (**C**), ATP leakage (**D**), intracellular protein concentration (**E**), and SDS-PAGE spectrum (**F**) of the tested strains. (*n* = 3, ****, ***, **, * and ns represent *p* < 0.0001, *p* < 0.001, *p* < 0.01, *p* < 0.05, and *p* > 0.05, respectively).

The effects of EOS-PL on the leakage of proteins and nucleic acids were shown in Figure 5A,B. Compared with the control group, treatment with 2 MIC EOS-PL significantly increased the OD_280_ and OD_260_ by 78.68% and 242.59% in *C. krusei* (*p* < 0.05), by 61.57% and 164.22% in *R. mucilaginosa* (*p* < 0.05), by 65.65% and 356.26% in *E. coli* (*p* < 0.05), and by 75.19% and 410.00% in *S. aureus* (*p* < 0.05), respectively.

AKPase is an enzyme located between the cell wall and membrane, and its activity can be used to evaluate cell wall damage in microbial suspensions. The results of this study are shown in Figure 5C. Compared with the control group, at EOS-PL concentrations of 2 MIC, MIC, and 1/2 MIC, the extracellular AKPase activity of *C. krusei* significantly increased by 356.99%, 258.17%, and 208.77%, respectively (*p* < 0.05). *R. mucilaginosa* significantly increased by 415.54%, 327.53%, and 138.91% (*p* < 0.05); *E. coli* increased by 298.29%, 199.40%, and 102.51%; and *S. aureus* significantly increased by 157.86%, 123.07%, and 71.27%, respectively (*p* < 0.05).

ATP is recognized as a key carrier for energy metabolism and physiological processes in microorganisms. A decrease in ATP levels indicates impaired mitochondrial function. The effects of EOS-PL on ATP leakage are presented in Figure 5D. Compared with the control group, when the EOS-PL concentration was 2MIC and MIC, the ATP leakage of *C. krusei* increased significantly by 818.21% and 293.32%, respectively (*p* < 0.05). When the concentration was 1/2 MIC, it increased by 65.08%. *R. mucilaginosa* increased significantly by 224.99% and 142.90% at concentrations of 2MIC and MIC, respectively (*p* < 0.05). When the concentration was 1/2 MIC, it increased by 26.06%. When the EOS-PL concentration was 2MIC, MIC, and 1/2 MIC, the ATP leakage of *E. coli* significantly increased by 350.46%, 275.25%, and 162.44%, respectively (*p* < 0.05). *S. aureus* significantly increased by 413.76%, 301.03%, and 147.68%, respectively (*p* < 0.05).

These results suggest that EOS-PL might impair the integrity and permeability of cell membranes and cell walls, trigger leakage of intracellular components, and could potentially result in cell death. The extent of damage was found to be concentration-dependent, which was consistent with the findings reported by Yuan et al. [34] that rose essential oil can cause content leakage in *P. putida*.

#### 3.4.7. Effect of EOS-PL on Intracellular Proteins

Proteins are the most important biological macromolecules supporting cell structure and viability. First, the intracellular protein content of microbial cells treated with different concentrations of the antimicrobial agent was determined in this study (Figure 5E). Based on this result, the effects of EOS-PL on intracellular proteins were further examined using SDS-PAGE (Figure 5F). As shown in Figure 5E, compared with the control group, the intracellular protein content of *C. krusei* was significantly reduced by 83.91%, 37.11%, and 23.20% at 2 MIC, MIC, and 1/2 MIC EOS-PL concentrations, respectively (*p* < 0.05). *R. mucilaginosa* was significantly reduced by 87.64%, 57.97%, and 35.52%; *E. coli* was reduced by 63.53%, 11.72%, and 8.55%; and *S. aureus* was reduced by 65.66%, 14.23%, and 1.28%, respectively. As illustrated in Figure 5F, compared to the control group, the number and intensity of protein bands in the treated groups were observed to decrease with increasing EOS-PL concentrations. For *C. krusei*, at 1/2 MIC, bands between 40 and 100 kDa were significantly lightened, and the 70 kDa band disappeared. At MIC, bands near 50 kDa and 73 kDa were further lightened. At 2 MIC, most bands were nearly absent. For *R. mucilaginosa*, at 1/2 MIC, bands between 40 and 100 kDa were lightened, with notable reductions at 100 kDa and 45 kDa. At MIC, bands continued to fade, and those near 100 kDa and 40–50 kDa were nearly absent. At 2 MIC, bands were almost entirely absent. For *E. coli*, at 1/2 MIC, bands at 45 kDa and 60–100 kDa were lightened. At MIC, bands between 60 and 100 kDa were further reduced and nearly disappeared. At 2 MIC, only bands between 30 and 60 kDa remained, with significantly reduced intensity. For *S. aureus*, at 1/2 MIC and MIC, bands at 45 kDa and 70–100 kDa were markedly lightened, with no significant differences between these two concentrations. At 2 MIC, bands between 60 and 100 kDa disappeared, and bands between 30 and 60 kDa were noticeably lightened.

These results indicate that a higher concentration of EOS-PL caused more severe protein damage, leading to enhanced antimicrobial efficacy. This may be attributed to the capacity of EOS-PL to supply protein synthesis, facilitate the degradation of high-molecular-weight proteins, and modify protein expression profiles, which appear to differentiate cellular growth and metabolism and potentially enhance its anti-cellular effectiveness.

### 3.5. Application of EOS-PL in the Preservation of Blue Honeysuckle Berries

The surface of fruits naturally harbors complex microbial communities, including bacteria, yeasts, and various fungal spores. In this study, native epiphytic microorganisms on the peel of blue honeysuckle berries would compete with target spoilage yeasts for nutrients and attachment sites, and even secrete antagonistic metabolites, which would severely interfere with experimental outcomes. NaClO features low cost, convenient operation and broad-spectrum bactericidal capacity; thus, it is widely adopted for cleaning and disinfection in the food industry [35]. Low-concentration NaClO is used to remove exogenous bacteria attached to fruit peels in the field, avoiding initial microbial background differences caused by uneven field pollution. Notably, low-concentration NaClO only acts on fruit epidermis and fails to eradicate tissue-colonized endogenous spoilage yeasts, so it does not hinder the evaluation of the targeted antimicrobial capacity of EOS-PL. Complete rinsing with sterile distilled water afterward eliminates residual chlorine and prevents undesired chemical interactions with the natural antibacterial composite system.

#### 3.5.1. Effect of EOS-PL on the Appearance and Color of Blue Honeysuckle Berries

**Figure 6:**
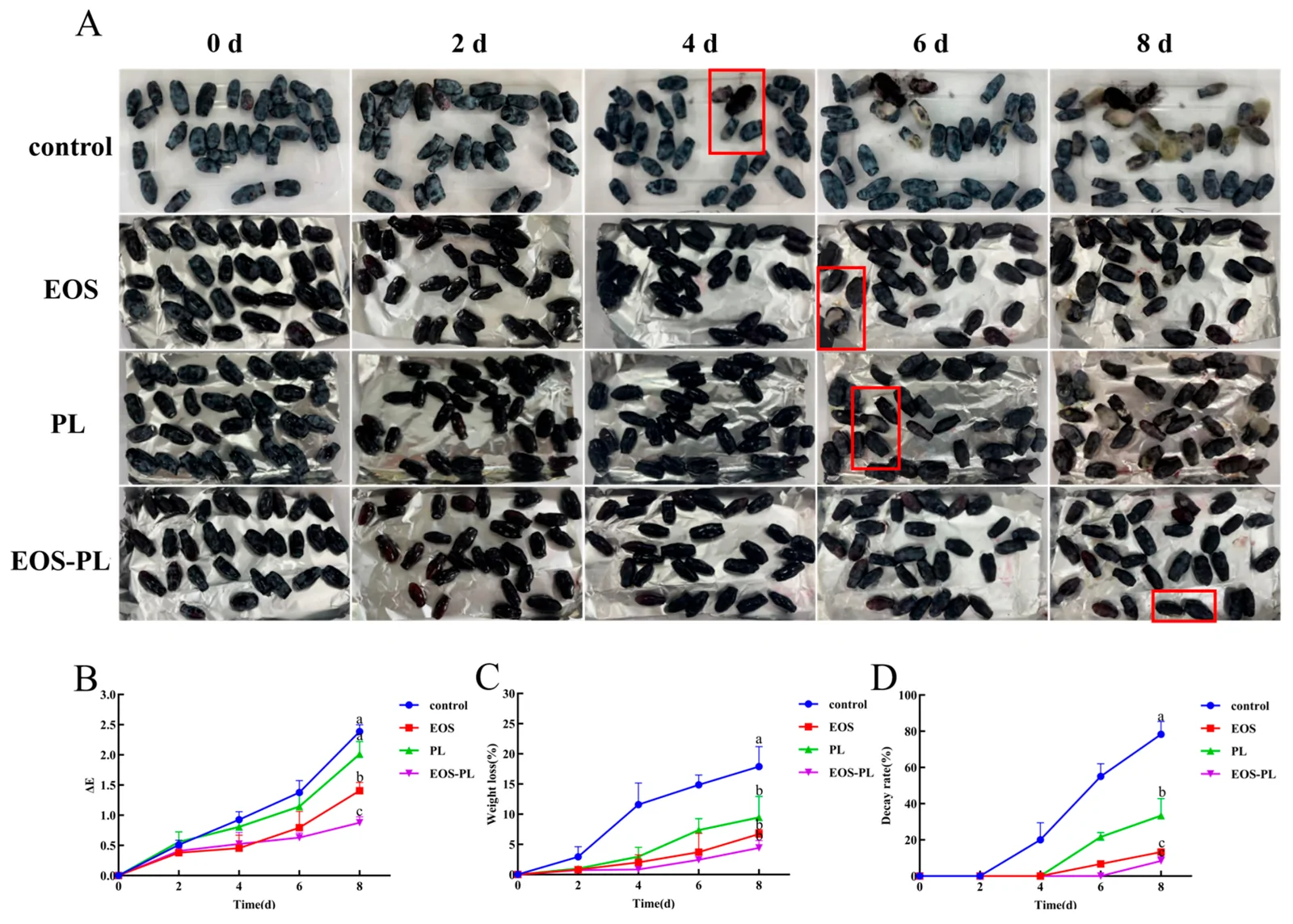
Effects of different preservatives on the appearance (**A**), color difference (**B**), weight loss rate (**C**), and decay rate (**D**) of blue honeysuckle berries. *n* = 3; lowercase letters (a–c) refer to statistically significant differences among the groups (*p* < 0.05). The red boxes highlight the decay of blue honeysuckle berries.

The visual appearance of blue honeysuckle berries under different treatments is shown in Figure 6A. Spoilage and decay were observed in the control group starting from 4 d, while browning and mold spots appeared in both the EOS and PL groups at 6 d. In contrast, microbial contamination was effectively suppressed in the EOS-PL group, extending the shelf life of the berries to 8 d. This demonstrated the strong synergistic antimicrobial effect of EOS and ε-PL.

Changes in the total color difference (ΔE) were evaluated by measuring the L*, a*, and b* values of the berries across treatment groups (Figure 6B). The ΔE was observed to increase gradually over time, which was attributed to pigment accumulation resulting from water loss and decay during storage, consistent with previous findings [36]. At 8 d, the ΔE for the control, EOS, and PL groups was recorded as 2.32, 1.67, and 2.06, respectively. The EOS-PL group exhibited the slightest color change, with a ΔE of only 0.93, suggesting that EOS-PL treatment more effectively inhibited oxidative browning and thus delayed color deterioration.

#### 3.5.2. Effect of EOS-PL on the Weight Loss and Decay Rate of Blue Honeysuckle Berries

During storage, blue honeysuckle berries gradually lose quality due to microbial contamination, respiratory metabolism, and water loss through transpiration. The effects of different treatments on the weight loss rate of blue honeysuckle berries are shown in Figure 6C. At 8 d, the weight loss rate of the control group reached 17.89%. Compared with the control group, the weight loss rates of the EOS group and PL group were significantly reduced by 62.49% and 47.18%, respectively, while that of the EOS-PL group was significantly reduced by 75.29% (*p* < 0.05).

The decay rate is an important indicator for evaluating the degree of microbial infection of fruits. The effects of different treatments on the decay rate of blue honeysuckle berries are shown in Figure 6D. With time, the decay rate of blue honeysuckle berries gradually increased. At 8 d, the decay rate of the control group reached 78.33%. Compared with the control group, the decay rates of the EOS group and PL group were significantly reduced by 82.98% and 57.45%, respectively, while that of the EOS-PL group was significantly reduced by 89.36% (*p* < 0.05). The results indicate that a physical barrier can be formed on the surface of blue honeysuckle berries by EOS and ε-PL. This barrier not only prevented the infection of harmful bacteria but also effectively inhibited the water loss and respiration of the berries, thereby exerting a better synergistic fresh-keeping effect.

#### 3.5.3. Effect of EOS-PL on pH and TSS Content of Blue Honeysuckle Berries

**Figure 7:**
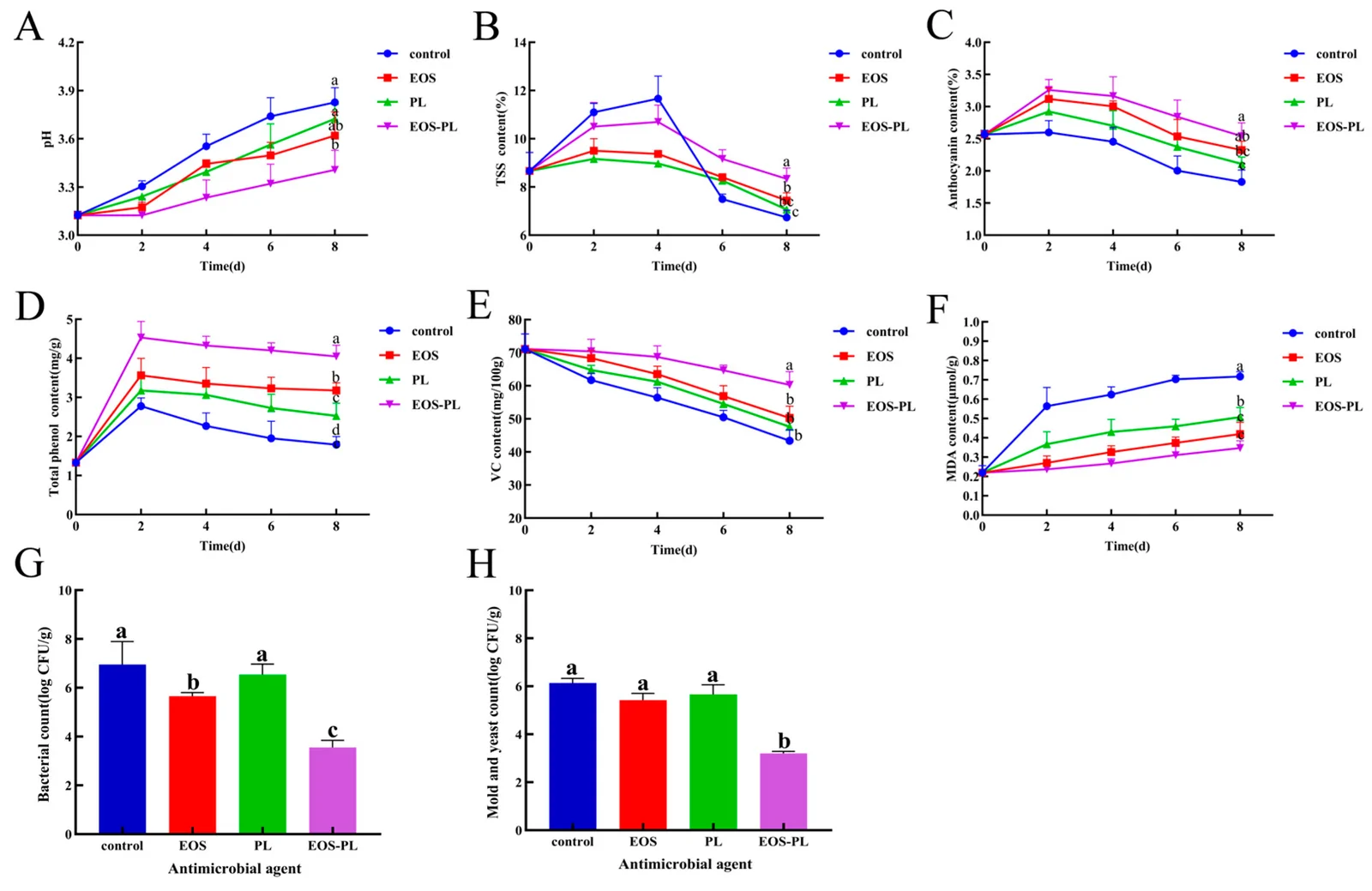
Effects of different preservatives on pH (**A**), TSS content (**B**), anthocyanin content (**C**), total phenolic content (**D**), VC content (**E**), MDA content (**F**), bacterial count (**G**), mold and yeast count (**H**) of blue honeysuckle berries. *n* = 3; lowercase letters (a–d) refer to statistically significant differences among the groups (*p* < 0.05).

The effects of different treatments on the pH of blue honeysuckle berries are shown in Figure 7A. With time, a gradual upward trend was observed in the pH. This was because the respiratory metabolism of blue honeysuckle berries was enhanced during the post-ripening process, leading to the gradual decomposition of organic acids [37]. The most pronounced change was recorded in the control group, with the pH rising to 3.83 by 8 d. The pH of the EOS and PL groups increased to 3.61 and 3.72, respectively, while the EOS-PL group exhibited the lowest pH value of 3.40. This indicated that EOS-PL treatment can more effectively inhibit the respiratory metabolism of blue honeysuckle berries during storage.

Total soluble solids (TSS), an indicator of sugar content in fruits that directly influences flavor, were measured as shown in Figure 7B. The TSS content generally exhibited a trend of first increasing and then decreasing, which was consistent with the findings described by Chang et al. [38]. This was because macromolecular carbohydrates such as starch were decomposed into soluble sugars during the ripening of blue honeysuckle berries, resulting in the accumulation of TSS. After the berries reached full ripeness, their respiratory activity was enhanced, and TSS were gradually consumed, leading to a decrease in their content [39]. At 8 d, compared with the control group, the TSS contents of the EOS and EOS-PL groups were significantly increased by 10.39% and 23.77%, respectively (*p* < 0.05). The PL group increased by 4.95%. This also indicates that EOS-PL treatment can reduce nutrient loss by inhibiting respiratory metabolism, thereby maintaining the berries’ original state and achieving a better fresh-keeping effect.

#### 3.5.4. Effect of EOS-PL on Anthocyanin, Total Phenols, and VC Content in Blue Honeysuckle Berries

Changes in anthocyanin and total phenolic content are shown in Figure 7C and Figure 7D, respectively. Both parameters exhibited an initial increase followed by a gradual decrease, which was attributed to the combined effects of fruit ripening during early storage and senescence in later stages. On 8 d, compared with the control group, the anthocyanin and total phenolic contents increased by 27.03% and 77.38% in the EOS group, 15.22% and 40.87% in the PL group, and 38.91% and 125.99% in the EOS-PL group, respectively. These results demonstrated that EOS-PL effectively delayed the senescence of blue honeysuckle berries, thereby enhancing preservation efficacy.

VC is a key factor for evaluating fruit quality and determining fruit freshness. Figure 7E showed the changes in VC content among different treatment groups, which generally exhibited a gradual downward trend. This was the result of the gradual accumulation of oxidative damage to blue honeysuckle berries during storage. On 8 d, the VC contents of the control, EOS, and PL groups were 43.38, 50.29, and 47.62 mg/100 g, respectively, while that of the EOS-PL group reached 60.27 mg/100 g. The VC content of the EOS-PL group was significantly higher than that of the other groups (*p* < 0.05), indicating that EOS-PL can slow down the loss of nutrients such as VC.

It could be concluded from the determination of anthocyanin, total phenol, and VC contents in blue honeysuckle berries that the antioxidant status of blue honeysuckle berries could be better maintained after EOS-PL treatment. This treatment also inhibited microbial growth and delayed the softening and decay processes, thereby effectively extending the shelf life of blue honeysuckle berries.

#### 3.5.5. Effect of EOS-PL on the MDA Content of Blue Honeysuckle Berries

During the ripening process of blue honeysuckle berries, the accumulation of ROS triggers lipid peroxidation, leading to MDA production and loss of membrane integrity [40]. As shown in Figure 7F, the most rapid increase in MDA content was observed in the control group, reaching 0.72 μmol/g at 8 d. The MDA contents of the EOS and PL groups were measured at 0.42 and 0.51 μmol/g, respectively, while the EOS-PL group exhibited the lowest value of 0.34 μmol/g. This was attributed to the ability of EOS-PL treatment to activate the oxidative defense system, thereby inhibiting lipid peroxidation in the late storage period, preserving membrane integrity, and delaying senescence and spoilage of the berries.

#### 3.5.6. Effect of EOS-PL on Microbial Indicators

The microbial count during the storage period of blue honeysuckle berries is an important indicator for evaluating the preservation effect. The results of the bacterial, mold and yeast counts are shown in Figure 7G,H. At the end of storage, the bacterial counts in the control group, EOS group, and PL group were 6.95, 5.65, and 6.53 log CFU/g, respectively, while the EOS-PL group was 3.55 log CFU/g, significantly lower than the other groups (*p* < 0.05). For mold and yeast, the numbers of the control group, EOS group, and PL group were 6.13, 5.42, and 5.66 log CFU/g, respectively, which were significantly higher than the 3.20 log CFU/g in the EOS-PL group (*p* < 0.05). A similar result was also shown in a previous study, where the addition of 4% cassava starch combined with cinnamon essential oil or clove essential oil coating exhibited a reduced number of microorganisms in papaya fruit; aerobic bacteria and mold and yeast decreased to 1.48 and 1.95 log CFU/g, respectively [41].

## 4. Conclusions

The results of this study demonstrated that blue honeysuckle berries could be preserved more effectively when antimicrobial strategies were designed based on dominant spoilage microorganisms rather than using a general broad-spectrum approach. The isolated spoilage yeasts, *C. krusei* and *R. mucilaginosa*, provided clear microbial targets for constructing the EOS-PL system based on *Litsea cubeba* essential oil, cinnamon essential oil, and ε-polylysine. The combined system exhibited stronger antimicrobial activity than the individual components, indicating a synergistic effect between essential oils and ε-polylysine. This effect may be largely linked to membrane injury triggered by essential oils, which appear to elevate cell permeability and promote interactions between ε-lysine and in traceability targets. Successful interactions could help maintain membrane integrity and cellular metabolism. Application to blue honeysuckle berries further confirmed that this targeted antimicrobial system effectively suppressed endogenous microbial growth, reduced decay, and delayed quality deterioration during storage. The treatment also helped maintain key quality attributes, including color, anthocyanin content, and vitamin C content. In summary, a design route for natural composite preservatives targeting spoilage yeasts was proposed in this study. The EOS-PL composite system exhibited promising potential in preserving the postharvest quality of blue honeysuckle under laboratory conditions. Nevertheless, large-scale commercial storage validation, delivery carrier optimization and systematic safety evaluations still need to be implemented before this system can be adopted for industrial use. The results obtained in the present work could provide preliminary laboratory references for the exploitation of targeted natural preservation strategies for berry fruits.

## Figures and Tables

**Table 1:** MIC of SA, EOS, and EOS-PL on the tested strains and the synergistic antimicrobial effect of EOS-PL.

Strains	MIC/mg·mL^−1^ (SA)			MIC/mg·mL^−1^ (LCEO:CEO)			MIC/mg·mL^−1^ (EOS:ε-PL)			FICI	Result
	LCEO	CEO	ε-PL	1:2	1:1	2:1	1:2	1:1	2:1		
*C. krusei*	1.000	0.500	2.000	0.250	0.500	0.500	0.500	0.500	0.125	0.500	Synergism
*R. mucilaginosa*	0.500	0.250	1.000	0.125	0.500	1.000	0.125	0.125	0.063	0.375	Synergism
*E. coli*	0.500	2.000	2.000	0.125	0.250	0.500	0.063	0.125	0.063	0.250	Synergism
*S. aureus*	1.000	1.000	2.000	0.500	1.000	1.000	0.125	0.250	0.125	0.500	Synergism

## Data Availability

The original contributions presented in the study are included in the article/Supplementary Materials. Further inquiries can be directed to the corresponding author.

## Supplementary Materials

The following supporting information can be downloaded at: https://www.mdpi.com/article/10.3390/foods15162784/s1, Table S1: PCR reaction system; Table S2: PCR cycling conditions; Table S3: Primer list; Table S4: Similarity results of Yeast C sequence; Table S5: Similarity results of Yeast R sequence; Table S6: Chemical composition of LCEO; Table S7: Chemical composition of CEO; Figure S1: Morphological identification result of strains; Figure S2: 26s rDNA PCR amplification electrophoresis of strains; Figure S3: Phylogenetic tree of strains.

## References

[1] Cheng Z., Bao Y.W., Li Z.Y., Wang J.X., Wang M.S., Wang S.H., Wang Y.Y., Wang Y.H., Li B. (2023). *Lonicera caerulea* (*Haskap* berries): A review of development traceability, functional value, product development status, future opportunities, and challenges. Crit. Rev. Food Sci. Nutr..

[2] Zhong X.J., Wang X., Zhou N., Li J.J., Liu J.C., Yue J.Y., Hao X.M., Gan M.L., Lin P.C., Shang X.Y. (2021). Chemical characterization of the polar antibacterial fraction of the ethanol extract from *Rosmarinus officinalis*. Food Chem..

[3] Kunová S., Sendra E., Hascík P., Vukovic N.L., Vukic M.D., Ben Hsouna A., Mnif W., Kacániová M. (2022). Microbiological Quality of Deer Meat Treated with Essential Oil *Litsea cubeba*. Animals.

[4] Su R.Y., Guo X.Y., Cheng S., Zhang Z.R., Yang H., Wang J.Z., Song L.Y., Liu Z.D., Wang Y.T., Lu X. (2023). Inactivation of *Salmonella* using ultrasound in combination with *Litsea cubeba* essential oil nanoemulsion and its bactericidal application on cherry tomatoes. Ultrason. Sonochem..

[5] Damasceno R.O.S., Pinheiro J.L.S., da Silva L.D., Rodrigues L.H.M., Emídio J.J., Lima T.C., de Sousa D.P. (2025). Phytochemistry and Anti-Inflammatory and Antioxidant Activities of *Cinnamomum osmophloeum* and Its Bioactive Constituents: A Review. Plants.

[6] Phan C., Do T., Thuong T. (2024). Two inclusion complexes of cinnamon essential oil and lemongrass essential oil with β-CD: Preparation, characterisation and effect on the preservation of mango fruits. Int. J. Food Sci. Technol..

[7] Shen J.Q., Zhao H.J., Zhang W.J., Qian Y.L., Zhang X.W., Liu Z.Y., Jia F. (2025). Exploring the antibacterial mechanism of *Zanthoxylum bungeanum* Maxim residue extract against *Shewanella baltica* and its application in refrigerated shrimp preservation. Food Control.

[8] Li Q.X., Yu S.N., Han J.Z., Wu J.L., You L.J., Shi X.D., Wang S.Y. (2022). Synergistic antibacterial activity and mechanism of action of nisin/carvacrol combination against *Staphylococcus aureus* and their application in the infecting pasteurized milk. Food Chem..

[9] Fan Q.X., Liu C., Gao Z.P., Hu Z.Q., Wang Z.L., Xiao J.B., Yuan Y.H., Yue T.L. (2021). Inactivation Effect of Thymoquinone on *Alicyclobacillus acidoterrestris* Vegetative Cells, Spores, and Biofilms. Front. Microbiol..

[10] Sun Y.A., Zhang M., Bhandari B., Bai B.S. (2021). Nanoemulsion-based edible coatings loaded with fennel essential oil/cinnamaldehyde: Characterization, antimicrobial property and advantages in pork meat patties application. Food Control.

[11] Zhao D.B., Wang S.D., Hu Y.S., Liu X., Tao J., Sagratini G., Xiang Q.S. (2022). Insight into the antibacterial activity of lauric arginate against Escherichia coli O157:H7: Membrane disruption and oxidative stress. LWT-Food Sci. Technol..

[12] Cui H.Y., Chen Y.Y., Aziz T., Al-Asmari F., Alwethaynani M.S., Shi C., Lin L. (2025). Antibacterial mechanisms of diacetyl on Listeria monocytogenes and its application in Inner Mongolian cheese preservation via gelatin-based edible films. Food Control.

[13] Liu X., Li Y.F., Wang S.D., Huangfu L.L., Zhang M.Y., Xiang Q.S. (2021). Synergistic antimicrobial activity of plasma-activated water and propylparaben: Mechanism and applications for fresh produce sanitation. LWT-Food Sci. Technol..

[14] Wang Z.L., Tian Y., Wang Q., Guo T.M., Yuan Y.H., Yue T.L., Jia H., Ge Q., Zhao Z.D., Cai R. (2023). Antimicrobial activity and mechanism of preservatives against *Alicyclobacillus acidoterrestris* and its application in apple juice. Int. J. Food Microbiol..

[15] Bai J.W., Li J.Q., Chen Z.Y., Bai X.D., Yang Z.Y., Wang Z.T., Yang Y. (2023). Antibacterial activity and mechanism of clove essential oil against foodborne pathogens. LWT-Food Sci. Technol..

[16] Tong D.Y., Han P.P., Li M.R., Wang Z.X., Zhao Z., Jia Y.M., Ning Y.W. (2025). Sucrose laurate and nisin synergistically inhibit Bacillus subtilis by multiple antibacterial targets and play promising application potential in bread preservation. Food Chem..

[17] Tian L., Ma L.Y., Tian Y.D., Zhou T., Zhang C.L., Pan H., Dai Y.N., Pu J.F., Fu C.Y., Zhang P.F. (2025). Antibacterial mechanism of lysine against Escherichia coli O157:H7, its action on lettuce and in reducing the severity of murine colitis. Food Microbiol..

[18] Ma C., Han Y.C., Liu R.L., Niu B., Shen C.Y., Chen H.Z., Yin M., Wu W.J., Gao H.Y. (2025). Effects of negative air ion treatment on postharvest storage quality and anthocyanin metabolism of grape. Food Chem..

[19] Yang B.Q., Han Y.C., Wu W.J., Fang X.J., Chen H.J., Gao H.Y. (2022). Impact of melatonin application on lignification in water bamboo shoot during storage. Food Chem.-X.

[20] Yi L.H., Qi T., Li X.F., Zeng K.F. (2024). Controlling soft rot of green pepper by bacteriocin paracin wx3 and its effect on storage quality of green pepper. Food Chem..

[21] Jahani R., Behnamian M., Dezhsetan S., Karimirad R., Chamani E. (2023). Chitosan nano-biopolymer/Citrus paradisi peel oil delivery system enhanced shelf-life and postharvest quality of cherry tomato. Int. J. Biol. Macromol..

[22] de Oliveira J.G., Albiero B.R., Calisto I.H., Bertolo M.R.V., Oldoni F.C.A., Egea M.B., Bogusz S., de Azeredo H.M.C., Ferreira M.D. (2022). Bio-nanocomposite edible coatings based on arrowroot starch/cellulose nanocrystals/carnauba wax nanoemulsion containing essential oils to preserve quality and improve shelf life of strawberry. Int. J. Biol. Macromol..

[23] Yang Y.J., Lin M.Y., Feng S.Y., Gu Q., Chen Y.C., Wang Y.D., Song D.F., Gao M. (2020). Chemical composition, antibacterial activity, and mechanism of action of essential oil from *Litsea cubeba* against foodborne bacteria. J. Food Process. Preserv..

[24] Shreaz S., Bhatia R., Khan N., Muralidhar S., Basir S.F., Manzoor N., Khan L.A. (2011). Spice oil cinnamaldehyde exhibits potent anticandidal activity against fluconazole resistant clinical isolates. Fitoterapia.

[25] Lee D.U., Park Y.J., Kang C.E., Cui C.H., Lee D.H., Lee N.K., Paik H.D. (2022). Synergistic antimicrobial activity of ε-polylysine, chestnut extract, and cinnamon extract against Staphylococcus aureus. Food Sci. Biotechnol..

[26] Liu Y.J., Wang R.L., Zhao L.L., Huo S.S., Liu S.C., Zhang H.X., Tani A., Lv H.X. (2022). The Antifungal Activity of Cinnamon-Litsea Combined Essential Oil against Dominant Fungal Strains of Moldy Peanut Kernels. Foods.

[27] Gao L., Hu Y., Sun M.L., Zheng X.F., Yang M., Rao S.Q. (2021). Synergistic antibacterial effects of carvacrol and ε-polylysine. Qual. Assur. Saf. Crops Foods.

[28] Liu M.M., Bai Y.N., Feng M.X., Wang X.D., Ni L.X., Cai L.Y., Cao Y.G. (2025). The synergistic antibacterial effects of allicin nanoemulsion and &-polylysine against Escherichia coli in both planktonic and biofilm forms. Food Chem..

[29] Liu F., Wang F.T., Du L.H., Zhao T., Doyle M.P., Wang D.Y., Zhang X.X., Sun Z.L., Xu W.M. (2018). Antibacterial and antibiofilm activity of phenyllactic acid against *Enterobacter cloacae*. Food Control.

[30] Meng L.Y., Ma J.Q., Liu C.H., Mao X.Z., Li J. (2022). The microbial stress responses of Escherichia coli and Staphylococcus aureus induced by chitooligosaccharide. Carbohydr. Polym..

[31] Boda S.K., Ravikumar K., Saini D.K., Basu B. (2015). Differential viability response of prokaryotes and eukaryotes to high strength pulsed magnetic stimuli. Bioelectrochemistry.

[32] Dai C.H., Yan P.F., Yin X.L., Shu Z.Z., Mintah B.K., He R.H., Ma H.L. (2025). Surfactin and its Antibacterial Mechanism on *Staphylococcus Aureus* and Application in Pork Preservation. Food Bioprocess Technol..

[33] Qi J.R., Xu H., Wu X.D., Lv Z.Y., Yue T.L., Yang H.H., Yuan Y.H. (2025). Antimicrobial activity and mechanism of protocatechualdehyde against *Alicyclobacillus* spp. And its effect on apple juice physicochemical properties. Food Control.

[34] Yuan Y.H., Liu L.X., Guo L., Wang L., Liu Y.G. (2023). Antibacterial mechanism of rose essential oil against *Pseudomonas putida* isolated from white *Hypsizygus marmoreus* at cellular and metabolic levels. Ind. Crops Prod..

[35] Wang D., Fletcher G.C., On S.L.W., Palmer J.S., Gagic D., Flint S.H. (2023). Biofilm formation, sodium hypochlorite susceptibility and genetic diversity of Vibrio parahaemolyticus. Int. J. Food Microbiol..

[36] Liu Y.T., Yuan Y., Duan S.Q., Li C., Hu B., Liu A.P., Wu D.T., Cui H.Y., Lin L., He J.L. (2020). Preparation and characterization of chitosan films with three kinds of molecular weight for food packaging. Int. J. Biol. Macromol..

[37] Li S.H., Luo S., Wang H., Liu H., Liu J.Y., Zhang X., Tian B.R. (2025). Chitosan/polyvinyl alcohol film loading β-acids/β-cyclodextrin inclusion complex: A shelf-life extension strategy for strawberry. Int. J. Biol. Macromol..

[38] Chang C.K., Tsai S.Y., Gavahian M., Cheng K.C., Hou C.Y., Yudhistira B., Lin S.H., Santoso S.P., Hsieh C.W. (2023). Direct and alternating current electric fields affect pectin esterase and cellulase in tomato (*Solanum lycopersicum* L.) fruit during storage. Postharvest Biol. Technol..

[39] Valverde-Miranda D., Díaz-Pérez M., Gómez-Galán M., Callejón-Ferre A.J. (2021). Total soluble solids and dry matter of cucumber as indicators of shelf life. Postharvest Biol. Technol..

[40] Smirnoff N. (2000). Ascorbic acid: Metabolism and functions of a multi-facetted molecule. Curr. Opin. Plant Biol..

[41] do Nascimento A., Toneto L.C., Lepaus B.M., Valiati B.S., Faria-Silva L., de Sao Jose J.F.B. (2023). Effect of Edible Coatings of Cassava Starch Incorporated with Clove and Cinnamon Essential Oils on the Shelf Life of Papaya. Membranes.

