# Supplementary material for: Membrane-Disruption-Enhanced Synergistic Antimicrobial Action of Essential Oils and ε-Polylysine for Targeted Inhibition of Spoilage Yeasts in Blue Honeysuckle (Lonicera caerulea L.)
Source: Foods. 2026 Aug 8;15(16):2784. doi: 10.3390/foods15162784 (PMC13512635; doi:10.3390/foods15162784)
Supplement: Supplementary file 1 [file foods-15-02784-s001.zip › foods-4401286-supplementary.pdf]

1. Morphological identification

Two types of yeast were isolated and purified, stained with methylene blue, and observed under an optical microscope.

2. Molecular biological identification

2.1. DNA extraction

Genomic DNA was extracted by Ezup column yeast genomic DNA extraction kit.

2.2. PCR amplification of 26S DNA D1/D2 region

PCR reaction system、PCR cycling conditions and Primer list were recorded in Tables S1, S2, and S3, respectively.

Table S1 PCR reaction system

Reaction components	Volume (μL)
10 X PCR Buffer	
dNTP (each 10 mM)	
Taq Plus DNA Polymerase（5 U/μL）	
50mM $MgSO_4$	total 12.5
Primer F (10 μM)	1
Primer R (10 μM)	1
Template (DNA)	1
ddH_2O	9.5
Total	25

Table S2 PCR cycling conditions

Temperature (°C)	Time	Program
95	5 min	
94	30 s	
57	30 s	30cycle
72	90 s	
72	10min	

Table S3 Primer list

Category	Name	Sequence 5 '-3	Amplified region	PCR length/bp
Yeast	NL1	GCATATCAATAAGCGGAGGAAAAG	26S rDNA D1/D2 region	around 500
	NL4	GGTCCGTGTTTCAAGACGG		

2.3. Gel electrophoresis

Following the amplification process, a reevaluation of PCR product integrity was conducted via 1.7 % agarose gel electrophoresis.

2.4. Purification and recovery of DNA gel

SanPrep column DNA gel recovery kit was used for Purification and recovery.

2.5. Sequence analysis

Sequence similarity analysis was conducted via BLAST alignment between the identified sequence and known homologous sequences retrieved from the GenBank database. Phylogenetic tree was constructed using MEGA 11.0.13 software.

3. Identification result

3.1. Morphological identification result

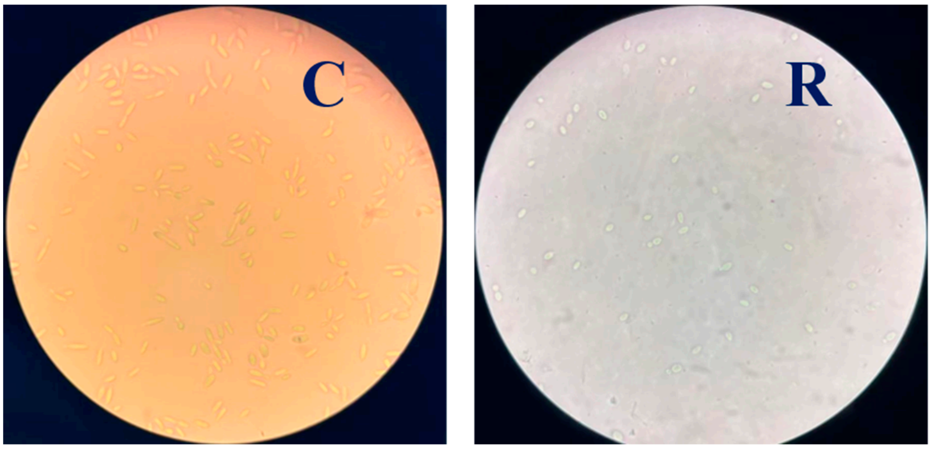

Figure S1. Morphological identification result of strains (C- *C. krusei,* R- *R. mucilaginosa*)

3.2. Molecular biological identification result

3.2.1. PCR amplification

PCR amplification result was shown in Figure S2.

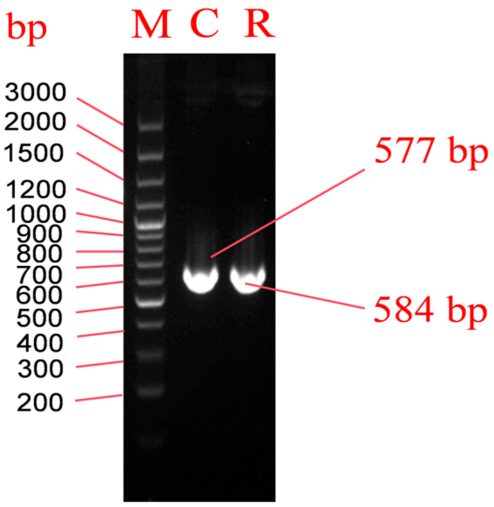

Figure S2. 26s rDNA PCR amplification electrophoresis of strains (M-Marker, C- *C. krusei,* R- *R. mucilaginosa*)

3.2.2. 26s rDNA sequence similarity results

3.2.2.1 Gene sequence

C. Krusei: GGAAAAGAAACCAACAGGGATTGCCTCAGTAGCGGCGAGTGAAGCGG CAAGAGCTCAGATTTGAAATCGTGCTTTGCGGCACGAGTTGTAGATTGCAGGTTGGAG TCTGTGTGGAAGGCGGTGTCCAAGTCCCTTGGAACAGGGCGCCCAGGAGGGTGAGAG CCCCGTGGGATGCCGGCGGAAGCAGTGAGGCCCTTCTGACGAGTCGAGTTGTTTGGG AATGCAGCTCCAAGCGGGTGGTAAATTCCATCTAAGGCTAAATACTGGCGAGAGACCG ATAGCGAACAAGTACTGTGAAGGAAAGATGAAAAGCACTTTGAAAAGAGAGTGAAAC

AGCACGTGAAATTGTTGAAAGGGAAGGGTATTGCGCCCGACATGGGGATTGCGCACCG
CTGCCTCTCGTGGGCGGCGCTCTGGGCTTTCCCTGGGCCAGCATCGGTTCTTGCTGCA
GGAGAAGGGGTTCTGGAACGTGGCTCTTCGGAGTGTTATAGCCAGGGCCAGATGCTGC
GTGCGGGGACCGAGGACTGCGGCCGTGTAGGTCACGGATGCTGGCAGAACGGCGCAA
CACCGCCCGTCT

R. mucilaginosa: GGAAAAGAAACTAACAAGGATTCCCCTAGTAGCGGCGAGCGAA
GCGGGAAGAGCTCAAATTTATAATCTGGCACCTTCGGTGTCCGAGTTGTAATCTCTAGA
AATGTTTTCCGCGTTGGACCGCACACAAGTCTGTTGGAATACAGCGGCATAGTGGTGA
GACCCCCGTATATGGTGCGGACGCCCAGCGCTTTGTGATACATTTTCGAAGAGTCGAGT
TGTTTGGGAATGCAGCTCAAATTGGGTGGTAAATTCCATCTAAAGCTAAATATTGGCGA
GAGACCGATAGCGAACAAGTACCGTGAGGGAAAGATGAAAAGCACTTTGGAAAGAG
AGTTAACAGTACGTGAAATTGTTGGAAGGGAAACGCTTGAAGTCAGACTTGCTTGCCG
AGCAATCGGTTTGCAGGCCAGCATCAGTTTTCCGGGATGGATAATGGTAGAGAGAAGG
TAGCAGTTTCGGCTGTGTTATAGCTCTCTGCTGGATACATCTTGGGGGACTGAGGAACG
CAGTGTGCCTTTGGCGGGGGTTTCGACCTCTTCACACTTAGGATGCTGGTGGAATGGC
TTTAAACGACCCGTCTT

3.2.2.2. Similarity results

The sequences described in 3.2.2.1 were subjected to similarity analysis using the GenBan k database of the National Center for Biotechnology Information (NCBI, https://www.ncbi.n lm.nih.gov/), and the similarity results were shown in Tables S4 and S5.

Table S4 Similarity results of Yeast C sequence

Strains	GenBank accession	Degree of similarity
Pichia kudriavzevii	KY108853.1	100%
Pichia kudriavzevii	KY107892.1	100%
Pichia kudriavzevii	MG245830.1	100%
Pichia kudriavzevii	PZ170115.1	100%
Pichia kudriavzevii	KF811416.1	100%
Pichia kudriavzevii	MF461004.1	100%
Pichia kudriavzevii	XR003834615.1	100%
Pichia kudriavzevii	KY108845.1	100%
Pichia kudriavzevii	CP093519.1	100%
Pichia kudriavzevii	LC021482.1	100%

Table S5 Similarity results of Yeast R sequence

Strains	GenBank accession	Degree of similarity
Rhodotorula mucilaginosa	MT705221.1	100%
Rhodotorula mucilaginosa	MT705217.1	100%
Rhodotorula mucilaginosa	PQ530235.1	100%
Rhodotorula mucilaginosa	MT705233.1	100%
Rhodotorula mucilaginosa	PV580979.1	100%
Rhodotorula mucilaginosa	KY109134.1	100%
Rhodotorula mucilaginosa	MT001273.1	100%
Rhodotorula mucilaginosa	KY109129.1	100%
Rhodotorula mucilaginosa	LC438617.1	100%
Rhodotorula mucilaginosa	KY109063.1	100%

3.2.3. Phylogenetic analysis

Phylogenetic tree was shown in Figure S3.

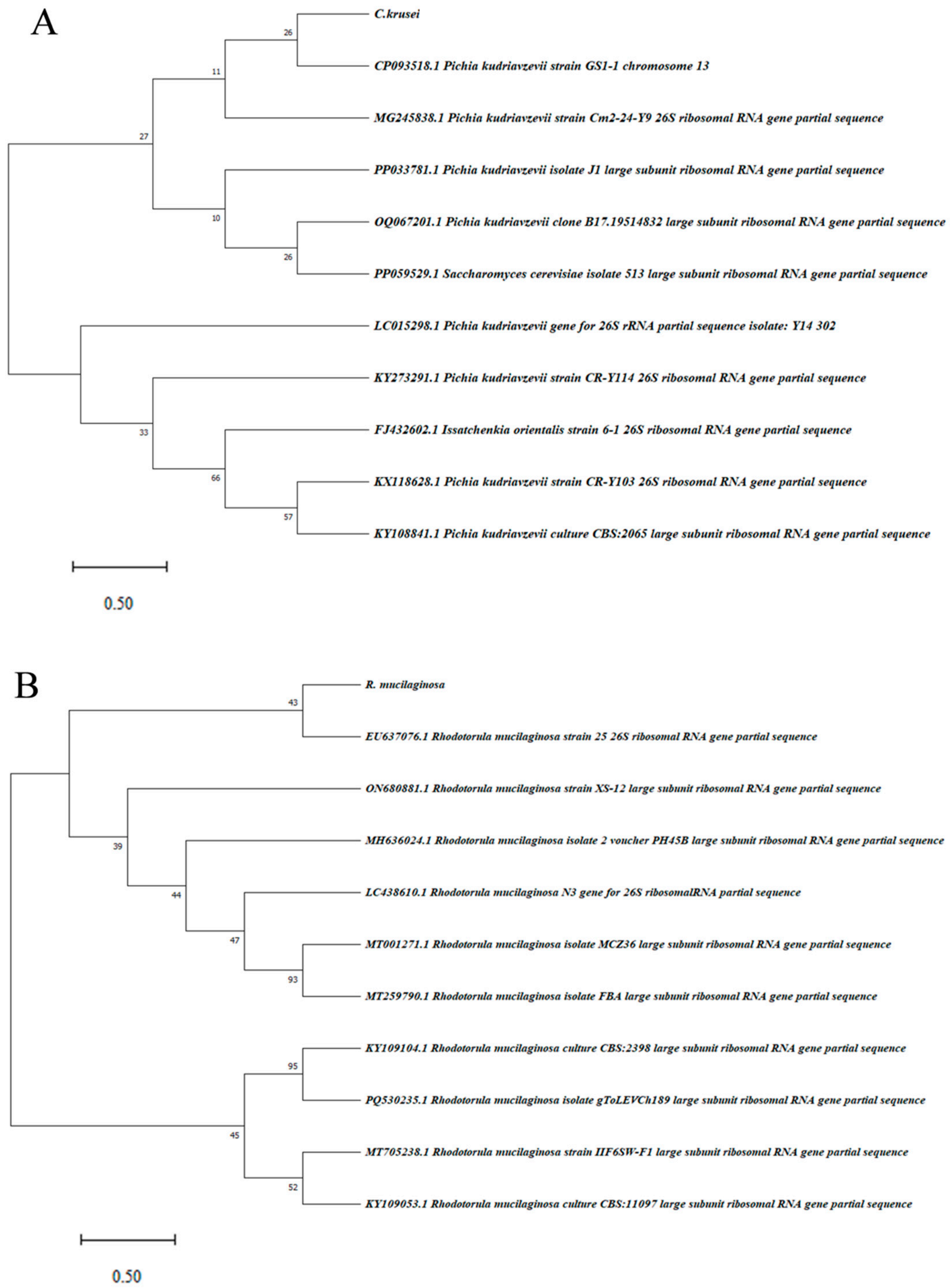

Figure S3. (A) *C. krusei* phylogenetic tree、(B) *R. mucilaginosa* phylogenetic tree

In summary, two types of spoilage yeast, *C. krusei* (*P. kudriavzevii*) and *R. mucilaginosa* were identified through morphological and molecular biology identification, respectively.

4. GC–MS analysis of essential oil

A total of 21 compounds (relative content > 0.1%) in LCEO were detected by GC–MS (Table S6). Geranial (39.94±0.38, %), Neral (36.48±0.33, %) and D-Limonene (11.03±0.19, %) were the predominant constituents.

Table S6 Chemical composition of LCEO

Serial Number	Retention Time (min)	Compound Name	Relative Content (%)
1	4.24	α-Pinene	0.81 ± 0.02
2	4.51	Camphene	0.23 ± 0.01
3	4.73	β-Pinene	0.84 ± 0.02
4	4.77	β-Myrcene	0.61 ± 0.01
5	4.90	γ-Terpinene	0.47 ± 0.01
6	4.93	p-Cymene	0.72 ± 0.02
7	5.12	1,8-Cineole	0.42 ± 0.01
8	5.39	D-Limonene	11.03 ± 0.19
9	5.76	6-Methyl-5-hepten-2-one	0.94 ± 0.03
10	6.16	Linalool	0.84 ± 0.02
11	6.42	Citronellal	0.56 ± 0.01
12	6.72	Terpinen-4-ol	0.34 ± 0.01
13	7.01	α-Terpineol	0.96 ± 0.03
14	7.33	Nerol	0.40 ± 0.01
15	7.66	Neral (β-Citral)	36.48 ± 0.33
16	7.94	Geranial (α-Citral)	39.94 ± 0.38
17	8.25	Geraniol	0.79 ± 0.02
18	9.18	Geranyl acetate	0.31 ± 0.01
19	13.46	β-Caryophyllene	0.51 ± 0.01
20	13.92	α-Humulene	0.26 ± 0.01
21	14.75	δ-Cadinene	0.30 ± 0.01
Total			97.76 ± 0.83

A total of 23 compounds (relative content > 0.1%) in CEO were detected by GC–MS (Table S7). (E)-Cinnamaldehyde (64.12 ± 0.35, %) and o-Methoxycinnamaldehyde (21.77 ± 0.06) were the predominant constituents.

Table S7 Chemical composition of CEO

Serial Number	Retention Time (min)	Compound Name	Relative Content (%)
1	3.76	Cyclofenchenene	0.16 ± 0.01
2	4.51	Benzaldehyde	1.25 ± 0.03
3	6.38	2-Hydroxy-benzaldehyde	0.28 ± 0.02
4	7.89	Phenylethyl alcohol	1.31 ± 0.02
5	9.18	Benzenepropanal	0.66 ± 0.01
6	9.30	endo-Borneol	0.22 ± 0.01
7	10.19	3-Phenylpropanol	0.29 ± 0.01
8	11.32	(E)-Cinnamaldehyde	64.12 ± 0.35
9	11.50	(Z)-Cinnamaldehyde	0.58 ± 0.01
10	11.69	3-Phenyl-2-Propen-1-ol	0.24 ± 0.01
11	12.58	α-Copaene	0.51 ± 0.02
12	13.46	Caryophyllene	0.16 ± 0.00
13	14.07	Acetic acid cinnamyl ester	4.53 ± 0.04
14	14.59	γ-Muurolene	0.20 ± 0.01
15	14.93	α-Curcumene	0.19 ± 0.02
16	15.16	α-Muurolene	0.18 ± 0.01
17	15.57	β-Bisabolene	0.13 ± 0.01
18	15.74	δ-Terpinene	0.36 ± 0.03
19	16.23	o-Methoxycinnamaldehyde	21.77 ± 0.06
20	17.41	Spathulenol	0.17 ± 0.01
21	17.89	Isoaromadendrene epoxide	0.16 ± 0.02
22	20.21	Coumarin	0.48 ± 0.02
23	22.47	Benzyl benzoate	0.15 ± 0.01
Total			98.10 ± 0.74
